# Effectiveness of Home-Based Cupping Massage Compared to Progressive Muscle Relaxation in Patients with Chronic Neck Pain—A Randomized Controlled Trial

**DOI:** 10.1371/journal.pone.0065378

**Published:** 2013-06-07

**Authors:** Romy Lauche, Svitlana Materdey, Holger Cramer, Heidemarie Haller, Rainer Stange, Gustav Dobos, Thomas Rampp

**Affiliations:** 1 Department of Internal and Integrative Medicine, Kliniken Essen-Mitte, Faculty of Medicine, University of Duisburg-Essen, Essen, Germany; 2 Immanuel Hospital Berlin, Department of Internal and Complementary Medicine, Berlin, Germany; Research and Development Corporation, United States of America

## Abstract

Chronic neck pain is a major public health problem with very few evidence-based complementary treatment options. This study aimed to test the efficacy of 12 weeks of a partner-delivered home-based cupping massage, compared to the same period of progressive muscle relaxation in patients with chronic non-specific neck pain. Patients were randomly assigned to self-directed cupping massage or progressive muscle relaxation. They were trained and asked to undertake the assigned treatment twice weekly for 12 weeks. Primary outcome measure was the current neck pain intensity (0–100 mm visual analog scale; VAS) after 12 weeks. Secondary outcome measures included pain on motion, affective pain perception, functional disability, psychological distress, wellbeing, health-related quality of life, pressure pain thresholds and adverse events. Sixty one patients (54.1±12.7 years; 73.8%female) were randomized to cupping massage (n = 30) or progressive muscle relaxation (n = 31). After treatment, both groups showed significantly less pain compared to baseline however without significant group differences. Significant effects in favor of cupping massage were only found for wellbeing and pressure pain thresholds. In conclusion, cupping massage is no more effective than progressive muscle relaxation in reducing chronic non-specific neck pain. Both therapies can be easily used at home and can reduce pain to a minimal clinically relevant extent. Cupping massage may however be better than PMR in improving well-being and decreasing pressure pain sensitivity but more studies with larger samples and longer follow-up periods are needed to confirm these results.

**Trial Registration:**

ClinicalTrials.gov NCT01500330

## Introduction

Chronic musculoskeletal pain syndromes, such as back and neck pain, are a major public health problem in all industrialized countries, with one in two people experiencing neck pain during their lives [1]. Neck pain is associated with both substantial work absenteeism [2] and significant disability in daily life [3].

Most treatment options for chronic neck pain have proven only moderately effective to date [4], [5]. This is especially true for complementary therapies, which are being under-represented in many therapeutic guidelines. The sole exceptions to the latter are acupuncture and chiropractic, which are recommended in German guidelines for chronic neck pain alongside physiotherapy with acknowledged limitations [6].

Treatment guidelines also emphasize that patients should be encouraged to use therapies they can easily apply themselves; as long as they perceive them as effective. This might include the application of therapeutic heat (e.g. heat pads [7], balneotherapy) or the use of progressive muscle relaxation after Jacobson (PMR) [8], a technique used to teach patients to relax muscles through a two-step process. In PMR, patients start to deliberately contract muscles and hold the tension; secondly they release all tension and focus on the sensation of relaxation. Regular practice will then help patients to recognize tension and to voluntarily relax affected muscles. Other not specified therapies with reasonable cost-benefit ratio are also recommended [6].

Cupping therapy is an ancient medical treatment, which uses suction on the skin [9], [10]. Several techniques are used, from traditional cupping, where skin incisions are made to allow blood and other body fluids to escape, to dry cupping and cupping massage, where no such incisions are made. Cupping may be beneficial for many pain conditions [11]; and recent pilot trials also have shown significant effects from cupping for patients with chronic non-specific neck pain [12]–[15]. Cupping is thought to act mainly by increasing local blood circulation and relieving painful muscle tension [16]. In cupping massage (CM), the effects of cupping and massage are combined, with the cupping glasses being moved over the skin surface after negative pressure has been created [9].

Not only PMR, but also CM, is easily learned for use in patients’ own homes and rather inexpensive. Although cupping massage from a physician in a clinical setting might be effective [12], [14], no research has yet been conducted into the effects of a home-based cupping massage program.

This study therefore aimed to test the efficacy of 12 weeks of a partner-delivered home-based cupping massage, compared to the same period of progressive muscle relaxation in patients with chronic non-specific neck pain.

## Methods

### Ethical Approval and Trial Registration

The original and translated protocol for this trial and supporting CONSORT checklist are available as supporting information; see Protocol S1 (German), Protocol S2 (English) and Checklist S1. This trial was conducted between and December 2011 and May 2012 in the Department of Complementary and Integrative Medicine in Essen, Germany. The study was approved by the ethics committee of the University Hospital Essen (approval number: 12–4358) and registered at ClinicalTrials.gov (registry number: NCT01500330), prior to patient recruitment.

### Design

This trial was a randomized controlled clinical trial with two parallel groups. After baseline measurement patients were randomized to either a cupping massage or a progressive muscle relaxation group and introduced to their assigned intervention. Trial measurements were repeated post-intervention, 12 weeks after randomization. All measurements were conducted by an investigator blind to patients’ group allocation.

### Patients

Patients were recruited via a local newspaper advertisement, with a research assistant screening interested people by phone to assess their eligibility. People who met the trial inclusion criteria were invited to attend a trial assessment session two weeks later. In the meantime, they were asked to keep a daily pain diary; bringing this diary with them to the assessment session. During the latter, a study physician explored patients’ medical histories and drug usage; examined their physical health and neurological function. The physician also checked patients’ medical records that they provided, e.g. laboratory findings or x-rays. If patients met the trial inclusion criteria, and did not fulfill any exclusion criteria, they were given detailed written information about the study and their written informed consent was obtained.

Trial participants were required to be aged 18–75 and to have experienced non-specific neck pain for at least the previous three months, for a minimum of five days a week. Their mean neck pain intensity was required to be 45 mm or more on a 100 mm visual analog scale (VAS) [17], where 100 mm was described as “the worst pain imaginable”. Therefor the patients’ diaries were checked and the average pain intensity of the past 2 weeks was calculated.

The trial exclusion criteria included neck pain caused by trauma, disc protrusion, whiplash, congenital deformity of the spine, spinal stenosis, neoplasm, inflammatory rheumatic disease, or active oncologic disease, affective disorder, addiction and psychosis. In addition, patients who were pregnant or who had had invasive treatment of the spine within the previous four weeks, or spinal surgery within the previous year were excluded. Finally, patients using opiates and long-term corticosteroid medication (>10 mg Prednisolon or equivalent), those who had started a new treatment for neck pain within the previous six weeks, or were planning to start such treatment within the following twelve weeks were also excluded.

### Randomization and Blinding

Patients were randomly assigned to one treatment group using a non-stratified block-randomization approach with randomly varying block lengths. The “ranuni” random number generator of the SAS/STAT ® software (SAS Inc., Cary NC, US) was used to generate random numbers. Sequentially numbered sealed envelopes containing patients’ treatment assignments were prepared by a statistician who was not involved in conducting the study. Following each baseline assessment, the trial coordinator opened the next lowest numbered envelope to reveal patient’s treatment assignment. During the active treatment phase, only the trial coordinator had contact with patients and knew of their group allocation. The trial coordinator was not involved in patients’ outcome assessments and the outcome assessor remained blind to patients’ group allocation throughout.

### Interventions

#### Cupping Massage (CM) group

These participants, and a partner, relative or friend, as appropriate, attended a one-hour practical workshop to learn how to use cupping massage. The clinic regularly runs such sessions to teach patients and their partners cupping massage methods for home use. The workshop was led by an experienced teacher and two assistants and it began with an overview of the history, indications and contraindications of cupping massage, followed by its technique, risks and possible side effects. Cupping massage was then demonstrated by the teacher and a patient volunteer. Patients, and those accompanying them, then practiced the cupping massage technique, with staff members providing feedback and suggestions for improvement as needed. Patients practiced until they felt competent to use the cupping massage technique unaided. All patients were given a cupping glass (Ø3.5 cm, Karl Hecht GmbH, Sondheim/Rhön, Germany), 200 ml of arnica massage oil (Weleda AG, Schwäbisch-Gmünd, Germany) and detailed written information to take home. Patients were advised that they could attend a further ‘refresher’ session, at any time during the trial, if they felt that they needed to. They were asked to contact the trial coordinator if they wished to attend such a session, but none did so. Patients were also asked to contact the trial coordinator if they experienced any adverse events. All patients were telephoned halfway through the study to promote compliance with the trial and its treatments. All patients further received an instruction sheet in German with a summary of treatment advices, see appendix 1.

Two treatment sessions per week of 10–15 minutes’ duration each at comfortable intensity were recommended. Patients were advised that cupping massage might cause petechiae and ecchymosis for several days; necessitating awareness in social settings such as swimming pools.

#### Progressive Muscle Relaxation (PMR) group

Progressive muscle relaxation is a systematic technique used to achieve a deep state of relaxation, developed by Edmund Jacobson [8]. It is widely used by patients with chronic pain conditions; however previous research indicates that relaxation techniques might not be better than usual care for chronic neck pain [18], [19]. In this trial, it was applied to prevent patients from dropping out of the trial due to loss of motivation, because otherwise they would have waited for 12 weeks without any intervention. It was also used as an attention control. Progressive muscle relaxation could also easily be learned and applied at home. Participants in this group attended a one hour session led by a psychologist experienced in delivering relaxation training. They were taught about the history of relaxation and the process of undertaking it. They then practiced a shortened version of the PMR technique. They went on to discuss issues related to this exercise and were given written information and a training CD. The CD, which contained short and long versions of the PMR technique, was designed by a large statutory German health insurance company to relieve muscle tension and improve general wellbeing [20].

Patients were asked to practice relaxation at home twice a week for 20 minutes a session and to record their practice in a diary. At the end of the trial, they were also offered a cupping massage workshop, a cupping glass (Ø3.5 cm, Karl Hecht GmbH, Sondheim/Rhön, Germany) and 200 ml of arnica massage oil (Weleda AG, Schwäbisch-Gmünd, Germany), as incentives to complete the study.

### Outcome Measures

The primary outcome measure was perceived pain, as recorded on a 100 mm Visual Analogue Scale (VAS) [21] at week 12. Secondary outcome measures included pain on motion, pain quality, functional disability, psychological distress, wellbeing, health-related quality of life and pressure pain sensitivity.

#### Questionnaires

To measure pain on motion, patients were asked to flex, extend, laterally flex and laterally rotate their necks to the left and right. The evoked pain was measured on a 100 mm VAS, for each direction. An average pain on motion score was then calculated from these data for each patient [12], [22]. Despite its frequent use in clinical studies this measure has not been validated.

Patients’ affective perception of pain was measured using the Pain Description List (SBL), a 12-item short form of the Pain Perception Scale (SES) [23]. The SBL is part of the validated German Pain Questionnaire (DSF) [24], [25]. It includes four items describing affective dimensions of pain. A sum score for this scale is then calculated, with the highest possible score being 12.

Patients’ functional neck-related disability was measured using the Neck Disability Index (NDI) [26] his 10-item questionnaire determines how patients see their neck pain affecting their daily activities. The maximum score is 50. Scores of less than four indicate no disability; 5–14 indicate mild disability, 15–24 moderate disability and 25–34 severe disability. Scores above 35 indicate complete perceived disability [27]. Trial patients were also asked to indicate the number of days that neck pain had interfered with their daily activities in the past three months, and the extent of this global interference, on a 100 mm VAS, to give further measures of their perceived disability.

Patients’ psychological distress was measured using the 14-item Hospital Anxiety and Depression Scale (HADS) [28] on dimensions of anxiety and depression. Each scale results in a maximum of 21 points, with scores over 8 indicating possible subclinical disorder. Psychological wellbeing was measured using the Questionnaire on the Assessment of Physical Wellbeing (FEW-16) [29]. This questionnaire comprises four subscales, each containing four items: stress resistance, ability to enjoy, vitality and inner peace.

Health-related quality of life was assessed using the Short Form 36 Health Survey Questionnaire (SF-36) [30]. This comprehensive 36-item questionnaire yields an 8-scale health profile as well as two component summaries of physical and mental health-related quality of life.

The results of additional measures of stress perception (Perceived Stress Questionnaire, PSQ-20) [31] and locus of control beliefs (Health Related Control Beliefs, GKÜ) [32], also used in the present study, will be reported elsewhere, together with qualitative data gathered after the study’s end.

#### Pressure pain sensitivity

Patients’ pressure pain thresholds (PPT) were measured using a digital algometer (Somedic AB, Hörby, Sweden) with a 1 cm^2^ probe. Pressure was applied, using this instrument, in increments of 40 kPa/s until patients indicated a perception of pain in addition to pressure. PPTs were determined where patients perceived maximal pain. They were also measured bilaterally at three anatomically predefined sites; over the levator scapulae muscle (medial to insertion on angulus superior scapulae), the descending part of the trapezius muscle (midway between C7 and the acromion process) and the semispinalis capitis muscle (distal to its origin and 2 cm from the midline) [12], [33], [34]. The averages of three measurements for each of these 7 locations were used in the analysis.

#### Daily log

All patients used a log to record the daily intensity of their pain (VAS), their medication, cupping massage or relaxation practices and any other concurrent treatments. To analyze patients’ medication usage, compliance and concurrent treatments the number of days where patients used pain medication, cupping or progressive muscle relaxation and physiotherapy was calculated for each week. Patients also noted weekly whether they obtained adequate relief from their neck pain, on a ‘yes/no’ basis (Adequate Relief Scale) [35].

#### Patients’ expectation

All patients rated their expectations that cupping massage and progressive muscle relaxation would be successful on a 100 mm VAS with 0 mm indicating not successful at all and 100 mm indicating highest possible expectation. For analyses only the expectation towards the assigned treatment was used.

#### Adverse events

All adverse events were recorded. Patients experiencing such events were asked to see the study physician to assess their import and initiate any necessary response.

### Sample Size Calculation and Statistical Analysis

A previous study of cupping massage for chronic non-specific neck pain [14] led current researchers to expect a statistically significant between group difference of −14.3 mm (Cohen’s d = 0.66) on the VAS. Given an effect size of d = 0.66, and a two-sided level 5% t-test, 76 patients would be needed to detect such a group difference with a statistical power of 80%. Researchers planned to include 84 patients in this trial; recognizing a potential loss of analytical power due to patient withdrawal.

After study start the study statistician was no longer available for analysis, therefore analysis had been conducted by the study coordinator. Final analysis plan and all analyses were conducted on an ‘intention-to-treat’ basis, including all patients who were randomized, regardless of whether they gave a full set of data or adhered fully to the study protocol. Missing data were completed with the value of the last available record (last observation carried forward), instead of using the Markov chain Monte Carlo multiple imputation method, as initially planned.

Baseline data comparability was ensured using Student’s t-tests for continuous data and x^2^ test for categorical data. Outcome data were analyzed using univariate analyses of covariance (ANCOVA) which modeled each post-treatment outcome as a function of treatment group (classified factor), patients’ expectations (linear covariate), and its respective baseline value (linear covariate).

Patients’ compliance with their allocated treatment regimes, pain levels, medication usage and concurrent treatments (daily logs) were analyzed using repeated measures ANCOVA with patients’ expectations as a linear covariate (for pain levels, medication usage, concurrent treatments). In case of significant interaction, exploratory post-hoc tests were applied without Bonferroni correction for multiple testing. All analyses were done using SPSS software (Version 20.0, IBM, Copenhagen), instead of SAS/STAT®-Software (SAS Institute Inc., USA), as initially planned.

## Results

### Patients

**Figure 1:**
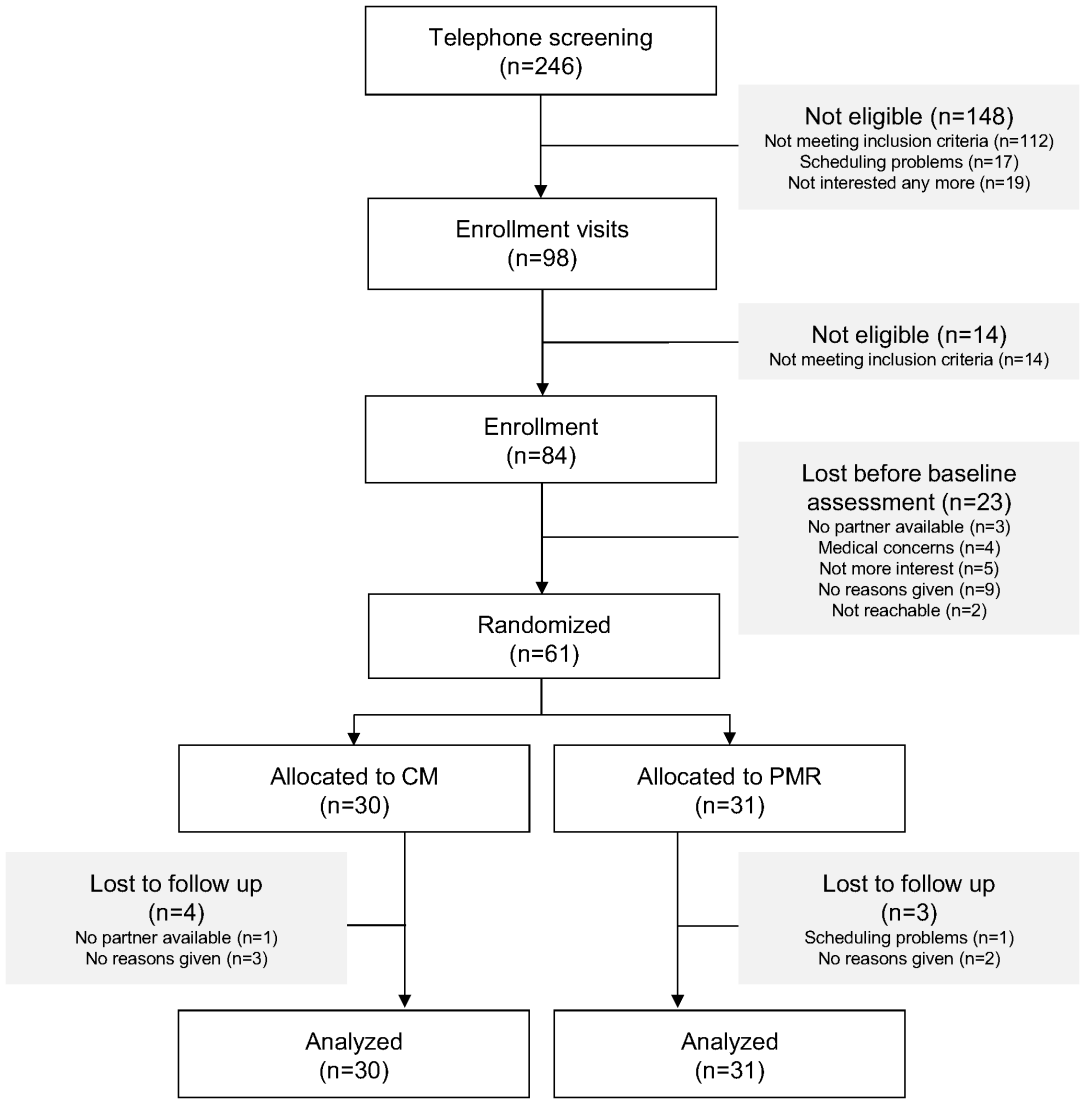
Consort flow chart of patient recruitment.

The Consort flowchart of patient recruitment is shown in the Figure 1. From 246 patients initially screened by telephone, 98 patients were seen by the study physician, of whom 84 were subsequently enrolled. The most common reasons for excluding patients were that they met one or more exclusion criteria, had scheduling problems and/or lost interest in the study. Of the 84 patients enrolled, 61 were randomized after baseline assessment. The 23 patients who withdrew at this stage had no study partner available, were no longer interested, had medical concerns, were not contactable after inclusion or withdrew without giving any reason. Seven of the 61 patients randomized were lost to follow-up; four in the intervention group (CM) and three in the control group (PMR). These patients did not attend for post-intervention assessment. Data for 30 cupping massage and 31 progressive muscle relaxation patients were finally analyzed.

Patients ranged in age from 24 to 74 years (Table 1). Most were in their mid-50′s and female. Patients with different levels of education and varied employment status were equally distributed in both groups. Patients reported an average of eight years of neck pain, with most having tried several types of past treatment. Most reported using pain medication only as necessary. No differences in patients’ major socio-demographic or pain-related characteristics were found between groups at baseline, although patients’ expectation that treatment would be successful was significantly higher in the CM group (Table 1).

**Table 1:** Socio-demographic and baseline characteristics for the study sample.

	Total (n = 61)	CM (n = 30)	PMR (n = 31)	P
**Socio-demographic characteristics**				
Age (years)	54.1±12.7	54.5±12.3	53.7±13.4	0.79
Gender (female/male in %)	73.8/26.2	80.0/20.0	67.7/32.2	0.21
BMI (kg/m^2^)	26.4±5.1	28.2±5.6	24.7±4.0	**0.008**
**Relationship status in %**				0.65
- Marriage/Partner	88.5	86.7	90.3	
- Single/divorced/widowed	11.5	13.3	9.7	
**Education in%**				0.10
-<High school	55.7	53.3	58.1	
- High school	21.3	13.3	29.0	
- University degree	23.0	33.3	12.9	
**Employment in %**				0.25
- Unemployed	54.1	46.7	61.3	
- Employed/self-employed	45.9	53.3	38.7	
**Neck pain characteristics**				
Duration of neck pain (years)	8.1±7.2	7.4±7.6	8.7±6.9	0.48
Pain intensity (mm VAS)	56.1±19.0	55.8±19.7	56.3±18.6	0.92
**Currently taking pain medication in %**				0.96
- Regularly	13.1	13.3	12.9	
- When needed	86.9	86.7	87.1	
**Treatments previously received in %**				
- Pain medication	57.4	53.3	61.3	0.53
- Injections	49.2	36.7	61.3	0.054
- Physiotherapy	57.4	43.3	71.0	**0.03**
- Massage	62.3	66.7	58.1	0.49
- Acupuncture	26.7	26.7	26.7	1.00
- Chiropractic	21.3	23.3	19.4	0.70
- Psychotherapy	18.0	13.3	22.6	0.35
- Relaxation	23.0	23.3	26.6	0.94
- Rehabilitation	16.4	20.0	12.9	0.45
Perceived ability to influence the own neck pain (mm VAS)	35.7±24.8	37.5±26.6	34.0±24.2	0.58
**Treatment expectancy (mm VAS)^a^**		81.8±17.5	62.3±31.0	**0.004**

Legend: ^a^Patients’ rated treatment expectancy for both treatments on a 100 mm VAS prior to randomization. Only treatment expectancy for the allocated treatment is shown.

### Compliance

**Figure 2:**
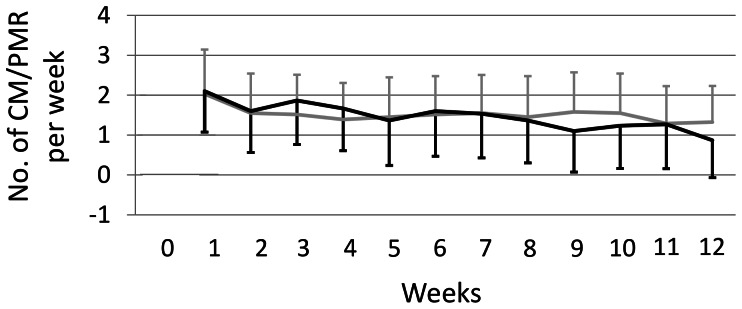
Patient compliance over the 12-week study period (mean and standard deviation). Legend: grey: PMR; black: CM.

The pattern of patients’ compliance for the 12 weeks of the trial can be seen in figure 2. Patients in the CM group used cupping massage on average 1.4±0.8 times a week. PMR was used 1.5±0.9 times a week on average by the control group. The repeated measurement analysis revealed a significant time×group interaction for compliance (p = 0.006). Post-hoc analysis showed significant time effects in the PMR group (p = 0.015) and the CM group (p = 0.011). Within the PMR group, the comparison of week 1 vs. weeks 3–12 was significant (all p<0.05), while in the CM group this was true for the comparison of week 1 vs. weeks 8–12 (all p<0.05). Patients in both groups were practicing their assigned treatment significantly more often in week 1 than from week 3 or week 8 respectively onwards. No further differences between the weeks were found. When comparing the groups at the single weeks, no significant differences at any week were found.

### Concurrent Treatments

**Figure 3:**
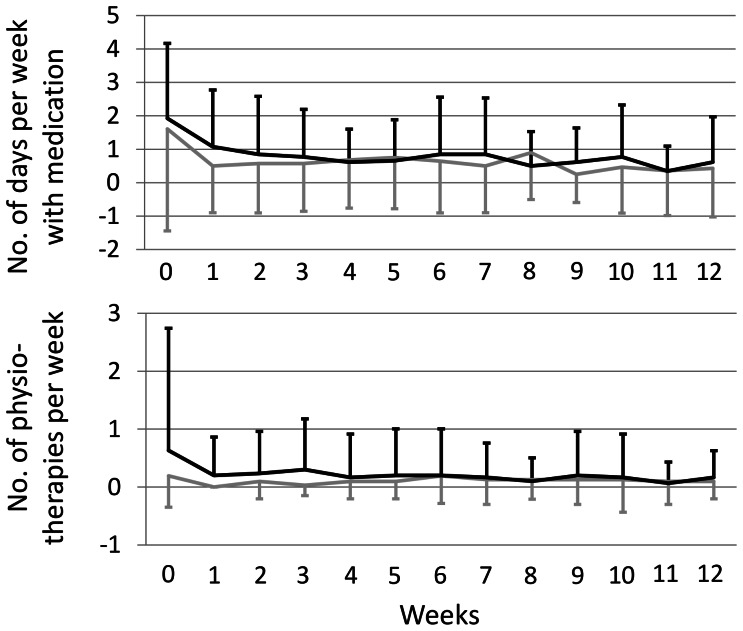
Patient’s concurrent medication use and physiotherapy treatments over the 12-week study period (mean and standard deviation). Legend: grey: PMR; black: CM.

The pattern of patients’ use of concurrent treatments for the 12 weeks of the trial can be seen in Figure 3. The CM group used medication at 0.8±1.0 days a week compared to 0.6±1.3 in the PMR group. Concurrent physiotherapy was received 0.2±0.6 times a week by the CM group and 0.1±0.2 times by the PMR group, however no significant differences between the groups could be observed.

### Outcome Measures

**Figure 4:**
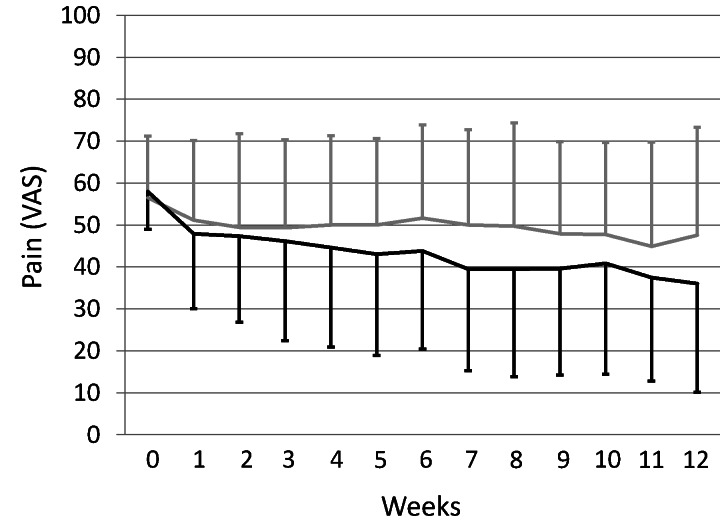
The pattern of patients’ pain over the 12-week study period (mean and standard deviation). Legend: grey: PMR; black: CM.

The course of pain ratings over the 12 weeks is shown in figure 4, the repeated measures ANCOVA revealed no significant effect of group allocation, but of time only (F_(3.8;767)_ = 9.43, p<0.001). In week 12, no significant group difference was found between the CM and the PMR groups regarding pain intensity on the VAS as the primary outcome measure (between-group difference −0.16 mm; 95% CI: −13.90 to 13.55 P = 0.98; Table 2).

**Table 2:** Patients’ pre- and post-intervention scores and estimated group differences at week 12.

	Cupping (30)		PMR (31)			
	Baseline	Week 12	Baseline	Week 12	Estimated groupdifference at week12 (95% CI)*	p
**Pain**						
Pain (VAS)	55.8±19.7	39.8±30.0	56.3±18.6	45.2±23.5	−0.16 (−13.90;13.55)	0.98
Pain at motion (VAS)	51.8±23.5	43.3±25.0	49.9±19.2	41.5±19.7	2.4 (−8.69; 13.47)	0.67
Affective-emotional painperception (SES)	3.2±2.9	2.9±3.9	3.0±3.5	2,5±3.1	0.37 (−1.37; 2.12)	0.67
**Disability**						
Disability (NDI)	15.5±4.3	12.6±5.2	17.9±4.9	16.8±5.1	−2.18 (−4.56; −0.21)	0.07
Days of interference in the past3 months	5.2±8.3	4.8±16.4	6.8±7.5	5.6±6.1	0.05 (−5.31; 5.41)	0.99
Interference with daily life (VAS)	31.4±21.2	25.7±23.8	32.7±23.4	24.2±19.3	5.26 (−6.70; 17.23)	0.38
**Psychological outcomes**						
Anxiety (HADS-A)	7.3±3.3	6.3±3.9	7.5±4.0	6.9±4.0	−0.54 (−1.87; 0.80)	0.42
Depression (HADS-D)	5.9±3.3	5.5±3.6	5.8±3.3	5.4±2.7	−0.05 (−1.21; 1.11)	0.93
Stress resistance (FEW16)	12.6±3.8	12.5±3.6	11.0±3.8	10.3±3.7	1.24 (−0.23; 2.71)	0.10
Ability to enjoy (FEW16)	12.5±3.6	12.5±3.6	12.5±3.7	11.9±3.4	0.24 (−1.10; 1.57)	0.72
Vitality (FEW16)	10.6±3.7	11.5±3.7	9.2±4.7	8.5±4.6	1.76 (0.01; 3.50)	**0.049**
Inner peace (FEW16)	11.3±4.5	11.7±4.4	9.5±4.6	9.0±4.3	1.60 (0.26; 2.94)	**0.02**
**Quality of life (SF-36)**						
Physical component summary	38.8±8.5	43.5±10.1	37.2±6.6	39.8±8.1	2.00 (−1.66; 5.66)	0.28
Mental component summary	47.0±11.5	45.9±12.8	47.5±11.6	46.6±11.6	−0.32 (−3.75; 3.11)	0.85
Physical functioning	64.2±22.7	71.2±22.5	70.3±18.5	73.2±18.1	1.97 (−3.64; 7.59)	0.49
Physical role functioning	46.7±40.3	56.7±40.4	44.35±42.2	46.8±41.7	10.02 (−8.25; 28.29)	0.28
Bodily Pain	40.7±12.9	53.9±21.2	36.4±12.4	41.8±15.9	6.26 (−3.28; 15.81)	0.19
General Health Perception	60.7±16.5	63.7±20.1	50.0±17.8	54.8±19.3	−0.56 (−7.59; 6.47)	0.87
Vitality	51.7±18.6	55.3±18.2	48.6±20.8	49.4±20.2	3.11 (−3.53; 9.75)	0.35
Social role functioning	71.7±19.9	75.0±19.4	70.6±25.9	75.0±23.7	−2.19 (−9.63; 5.25)	0.56
Emotional role functioning	63.3±41.2	56.7±44.8	73.1±38.9	71.0±39.2	−11.08 (−28.51; 6.36)	0.21
Mental health	63.9±17.2	66.1±21.1	63.9±16.9	61.4±16.7	5.37 (−0.36; 11.10)	0.07
**Pressure Pain Thresholds**						
Site of maximal pain	287.3±118.0	332.7±145.6	287.3±158.6	254.8±133.0	63.95 (6.33; 121.56)	0.03
Left levator scapulae muscle	343.6±171.0	412.8±159.7	296.0±148.8	294.3±146.5	92.43 (30.96; 153.90)	0.004
Right levator scapulae muscle	335.2±155.8	382.7±163.1	273.4±151.0	297.4±165.9	42.73 (−23.49; 108.95)	0.20
Left trapezius muscle	273.7±121.4	315.0±132.8	229.0±114.8	239.8±114.7	49.29 (−0.08; 98.66)	0.05
Right trapezius muscle	271.2±106.5	320.3±132.1	234.0±153.2	244.9±116.8	51.30 (1.31; 101.29)	0.044
Left semispinalis capitis muscle	219.5±93.8	249.6±107.2	178.9±84.9	210.1±91.6	9.88 (−25.71; 45.48)	0.58
Right semispinalis capitis muscle	217.9±91.1	256.7±97.5	186.7±120.5	196.1±90.5	59.07 (17.51; 100.62)	0.006

Legend: *Estimation results from the ANCOVA with baseline and expectation as covariates.

No between group differences were found for patients’ pain on motion, affective pain perception or disability (Table 2). Other disability measures (days of interference caused by pain, interference with daily life), measures of psychological distress (HADS) and quality of life (SF-36) did not show any significant group differences. Significant differences in favor of the CM group were only found for vitality and inner peace as part of the Questionnaire on the Assessment of Physical Wellbeing (both p<0.05). While the CM group increased their reported wellbeing on these scales, the PMR group’s scores decreased.

**Figure 5:**
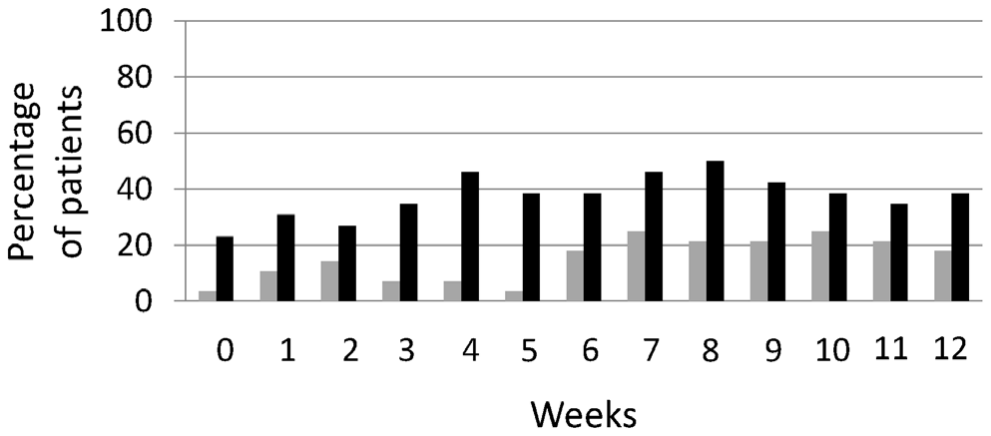
The pattern of patients’ perception of adequate relief over the 12-week study period (percentage of patients reporting adequate relief). Legend: grey: PMR; black: CM.

Patients in the CM group also showed higher pressure pain thresholds, with less sensitivity at four of seven sites, including the site of maximal pain (Table 2). Altogether the percentage of patients reporting adequate relief did not differ between the groups at week 12 (p = 0.09), see figure 5.

### Adverse Events

Three patients in the CM group reported adverse events during the trial, one of which was considered serious. One patient felt increased muscular tension and pain the morning after cupping massage, but this situation resolved some hours later. Another patient noted increased pain in the shoulder area, but cited a long history of shoulder problems following the use of crutches for a previous knee operation. This patient could not say if this pain was due to the massage intervention. A third patient was diagnosed with a prolapsed intervertebral disc. This serious adverse event was not considered a consequence of cupping massage. No side effects were reported in the PMR group.

## Discussion

Patients’ adoption of self-help strategies is an important goal in treating chronic musculoskeletal disorders, but studies of such interventions for patients with chronic neck pain are urgently needed. Although cupping massage and progressive muscle relaxation might provide such strategies, this is the first study to investigate these interventions’ effectiveness in a non-clinical setting.

This randomized controlled trial found 12 weeks’ home use of cupping massage was no more effective than progressive muscle relaxation in reducing participants’ pain and improving their functional impairment and quality of life. Differences were, however, found for some aspects of changes to their wellbeing and pressure pain sensitivity.

These results are mostly in line with past studies on cupping in chronic non-specific neck pain [12]–[15] which found single traditional cupping [15] or five applications of different types of dry cupping [12]–[14] better than waiting-list controls; comparable with the observed effects in the present study. Because the effect in the progressive muscle relaxation group was almost as large as that in the cupping massage group, no significant group difference was found. Both of the changes found were within the range of clinical significance for the VAS [36], however they are to be classified as minimal or little improvements only.

Interestingly, the effect sizes found within the cupping massage group, in the present study, compared closely to those found in past studies, despite major changes to the study design. While past studies’ treatments were delivered by professionals in clinical environments, the present study used home-based treatment. The present study also had a longer treatment interval (12 weeks) rather than the single treatment [15] or five treatments over two week patterns adopted elsewhere [12]–[14].

Potential modes of action of the cupping massage may involve increased local microcirculation; thought to reverse hypersensitivity. Hypersensitivity is seen mainly as a consequence of peripheral sensitization [27] and inflammation of local neural tissue [33] caused by an ischemia due to muscle spasm [37]. Increased pressure pain sensitivity is common in patients with chronic neck pain [33], [38], appearing in such muscles as the levator scapulae, trapezius and semispinalis capitis [33], [34]. The reduced hypersensitivity found at some areas might suggest that the microcirculation in patients’ neck areas was improved; however the results are too inconclusive to draw final conclusions.

Limitations of this study include different patients’ expectations of treatment effectiveness, which were entered as a covariate into the analyses, controlling for the influence of this variable.

A further limitation comes from patients’ high withdrawal rate prior to randomization that may have resulted in an underpowered study. Given the observed effects even the original sample size would not have been enough to detect differences in pain intensity. Furthermore, since most of the patients withdrew before randomization, the risk of a biased sample might also be very small.

Limitations may also arise from unknown differences between the partner-administered cupping massage treatment and relaxation exercises delivered solely by CD. A lack of a long-term follow-up and the impossibility of blinding patients and workshop providers to patients’ treatment allocations, are also possible sources of bias.

The study’s strengths include the adaptation of existing trial designs to investigate cupping massage and muscle relaxation in a non-clinical setting; providing results that are easily applied to everyday life. Its other strength is its use of a randomized controlled design with blinded outcome assessors, which is, however, only relevant for physiological outcome measures. The inclusion of an active control group practicing a relaxation method recommended by German neck pain guidelines [6], as well as the between group comparability of patients’ compliance, medication use and concurrent treatments are also strengths of the study.

In conclusion, cupping massage is no more effective than progressive muscle in reducing chronic non-specific neck pain. Both therapies can be easily used at home and can reduce pain to a minimal clinically relevant extent. Cupping massage may however be better than PMR in improving well-being and decreasing pressure pain sensitivity but more studies with larger samples and longer follow-up periods are needed to confirm these results.

## Supporting Information

Protocol S1 **Trial protocol.** Original study protocol in German as submitted to the ethics committee of the University Hospital Essen (approval number: 12–4358).
(DOCX)

Protocol S2 **Trial protocol.** English translation of the study protocol submitted to the ethics committee of the University Hospital Essen.
(DOCX)

Checklist S1 **CONSORT Checklist.**
(DOC)
