# Supplementary material for: Effectiveness of Home-Based Cupping Massage Compared to Progressive Muscle Relaxation in Patients with Chronic Neck Pain—A Randomized Controlled Trial
Source: PLoS One. 2013 Jun 7;8(6):e65378. doi: 10.1371/journal.pone.0065378 (PMC3676414; doi:10.1371/journal.pone.0065378)
Supplement: Protocol S1 — Trial protocol. Original study protocol in German as submitted to the ethics committee of the University Hospital Essen (approval number: 12–4358). (DOCX) [file pone.0065378.s001.docx]

Studienunterlagen zur Einreichung bei der Ethikkommission der Universität Essen

**Titel des Projekts:**

Randomisierte kontrollierte Studie zur Wirksamkeit der Schröpfkopfmassage als Heimanwendung bei chronischen Nackenschmerzen

Universität Duisburg-Essen

Alfried Krupp von Bohlen und Halbach - Stiftungsprofessur für Naturheilkunde

Kliniken Essen-Mitte

Klinik für Naturheilkunde und Integrative Medizin

Knappschaftskrankenhaus

Am Deimelsberg 34a

45276 Essen

**Leiter der Abteilung**

Prof. Dr. med. Gustav Dobos

Kliniken Essen-Mitte

Am Deimelsberg 34a

45276 Essen

Tel.: 0201 - 174 25001

Fax: 0201 - 174 25000

**Verantwortlicher Leiter und Studienarzt**

Dr. med. Thomas Rampp

Kliniken Essen-Mitte

Am Deimelsberg 34a

45276 Essen

Tel.: 0201 - 174 25013

Fax: 0201 - 174 25000

**Kooperationspartner**

Dr. med. Rainer Stange

Immanuel Krankenhaus Berlin (Standort Berlin-Wannsee)

Königstraße 63

D-14109 Berlin

Tel.: 030 - 80505 - 690

Fax: 030 - 80505 - 288

**Projektkoordination**

Dipl.-Psych. Romy Lauche

Kliniken Essen-Mitte

Am Deimelsberg 34a

45276 Essen

Tel.: 0201 - 174 25054

Fax: 0201 - 174 25000

**Biometrie und statistische Auswertung**

Dipl.-Stat. Rainer Lüdtke

Karl und Veronica Carstens-Stiftung

Am Deimelsberg 36

45276 Essen

Tel.: 0201 - 5630516

Fax: 0201 – 563050

Hiermit bestätige ich, dass ich den Prüfplan zur Kenntnis genommen habe und mit den Regelungen einverstanden bin, dass alle den Prüfplan betreffenden Fragen geklärt sind und dass ich die klinische Prüfung entsprechend diesem Prüfplan durchführen werde.

**Leiter der Abteilung**

_________________________________ _________________________________

Ort, Datum Prof. Dr. med. Gustav Dobos

**Leiter der Studie**

_________________________________ _________________________________

Ort, Datum Dr. med. Thomas Rampp

**Kooperationspartner**

_________________________________ _________________________________

Ort, Datum Dr. med. Rainer Stange

**Studienkoordination**

_________________________________ _________________________________

Ort, Datum Dipl.-Psych. Romy Lauche

**Biometrie**

_________________________________ _________________________________

Ort, Datum Dipl.-Stat. Rainer Lüdtke

Gliederung

[1. Prüfplanübersicht 5](#_Toc303856651)

[2. Zusammenfassung 8](#_Toc303856652)

[3. Wissenschaftlicher Hintergrund 8](#_Toc303856653)

[4. Studienziel 10](#_Toc303856654)

[5. Studiendesign 10](#_Toc303856655)

[6. Patienten 10](#_Toc303856656)

[6.1. Rekrutierung 10](#_Toc303856657)

[6.2. Einschlusskriterien 11](#_Toc303856658)

[6.3. Ausschlusskriterien meine kritik s. Zusammenfassung 11](#_Toc303856659)

[6.4. Einschlussuntersuchungen 12](#_Toc303856660)

[6.5. Randomisierung 12](#_Toc303856661)

[7. Intervention 12](#_Toc303856662)

[7.1. Schröpfkopfmassage 13](#_Toc303856663)

[7.2. Entspannungsübung 13](#_Toc303856664)

[8. Methoden 14](#_Toc303856665)

[8.1. Messmethoden 14](#_Toc303856666)

[8.2. Messzeitpunkte 15](#_Toc303856667)

[9. Zielparameter 16](#_Toc303856668)

[9.1. Primäre Zielgröße 16](#_Toc303856669)

[9.2. Sekundäre Zielgrößen 16](#_Toc303856670)

[9.3. Weitere Studienparameter 16](#_Toc303856671)

[10. Statistik 17](#_Toc303856672)

[10.1. Fallzahlschätzung 17](#_Toc303856673)

[10.2. Statistische Auswertung 17](#_Toc303856674)

[11. Nutzen/Risiko-Abwägung 18](#_Toc303856675)

[11.1. Schröpfkopfmassage 18](#_Toc303856676)

[11.2. Entspannungsübung 18](#_Toc303856677)

[11.3. Untersuchungsmethoden 18](#_Toc303856678)

[11.4. Zusammenfassende Nutzen/Risiko-Abwägung 18](#_Toc303856679)

[12. Studienprotokollerstellung und Ethikantrag 19](#_Toc303856680)

[13. Patientenversicherung 19](#_Toc303856681)

[14. Patienteninformation, Einwilligung und Datenschutz 19](#_Toc303856682)

[15. Finanzierung und wirtschaftliche Interessen 20](#_Toc303856683)

[16. Studienabbruch 20](#_Toc303856684)

[17. Eignung der Prüfstelle und wissenschaftlichen Einrichtung 20](#_Toc303856685)

[18. Literatur 20](#_Toc303856686)

[19. Anlagen 22](#_Toc303856687)

1. Prüfplanübersicht

| **Akronym** | NaSK |
| --- | --- |
| **Titel:** | Randomisierte kontrollierte Studie zur Wirksamkeit der Schröpfkopfmassage als Heimanwendung bei chronischen Nackenschmerzen |
| **Fragestellung:** | In dieser Studie soll die Wirksamkeit der Schröpfkopfmassage als Heimanwendung im Vergleich zu einem Entspannungstraining bei chronischen unspezifischen Nackenschmerzen getestet werden. Dazu werden die Schmerzintensität (VAS), bewegungsinduzierter Schmerz (PRTM), die Schmerzqualität (SBL), nackenschmerzspezifische Beeinträchtigungen (NDI), Wohlbefinden (FEW16), Stressempfinden (PSQ20) Lebensqualität (SF-36), Ängstlichkeit und Depressivität (HADS) sowie Kontrollüberzeugung (GKÜ) erfasst. Weiterhin wird die Druckschmerzempfindlichkeit (PPT) an definierten Punkten gemessen.  In einem Tagebuch werden die Schmerzhäufigkeit und die Einnahme von Medikamenten erfragt. Die persönlichen Erfahrungen, beobachtete Veränderungen und der Einfluss auf die Partnerschaft werden zudem in einer qualitativen Befragung am Ende der Behandlung erfasst.  Neben der Wirksamkeit wird auch die Sicherheit des Verfahrens evaluiert. |
| **Studiendesign:** | Randomisierte kontrollierte monozentrische Interventionsstudie mit 2 Gruppen:   - Schröpfkopfmassage als Heimanwendung. Anleitung in einem Workshop, danach 2x wöchentliche Anwendung über 3 Monate. - Entspannung als Heimanwendung. Anleitung in einem Workshop, danach 2x wöchentliche Anwendung über 3 Monate. Nach Beendigung der Studie wird die Teilnahme am Workshop zur Schröpfkopfmassage angeboten.   Es erfolgt eine Verblindung des Assessors. |
| **Studienzentrum:** | Kliniken Essen-Mitte, Klinik für Naturheilkunde und Integrative Medizin, Knappschafts-Krankenhaus |
| **Patientenzahl:** | 42 Patienten pro Gruppe, d.h. insgesamt 84 Patienten bei 2 Gruppen. Die Fallzahlschätzung erfolgte anhand des Antrags 09-3987. |
| **Studiendauer:** | Pro Patient 4 Monate, 6 Monate für die gesamte Studie |
| **Einschlusskriterien und Indikationen:** | - Alter zwischen 18 und 75 Jahren - Chronische unspezifische Nackenschmerzen seit mindestens 3 Monaten mit einer Intensität > 45mm auf der VAS - Vorhandensein eines Partners (Familienangehörige, Freunde, etc.), welche regelmäßig die erforderlichen Behandlungen durchführen können - Befunde vom Facharzt sind vorzulegen |
| **Ausschlusskriterien:** | - Spezifische Nackenschmerzen aufgrund: - Entzündlicher muskuloskeletaler Erkrankungen - Neurologischer Erkrankungen - HWS-Prolaps - Vorangegangene relevante Traumen (Frakturen, operationsbedingt) - Zustand nach Neoplasmen im Bereich der Wirbelsäule - Angeborene Fehlbildung der Wirbelsäule (Ausnahme: Leichte Skoliose) - Hauterkrankungen / Entzündungen in den zu behandelnden Arealen / allergische Hautveränderungen - Hämophilie / Antikoagulation / Thrombozytenaggregationsstörung - Schwere psychiatrische Erkrankung (z.B. Depression, Suchterkrankung, Schizophrenie) - Schwere komorbide somatische Erkrankung (z.B. Diabetes mellitus mit bestehender Polyneuropathie, onkologische Erkrankung ohne Remission) - Langzeitmedikation mit Corticosteroiden >10mg Prednisolon-Äquivalent - Therapie mit Opiaten - Neu begonnene oder aktuell modifizierte Medikation mit Einflüssen auf muskuloskeletale Schmerzen, z.B. Muskelrelaxantien, Psychopharmaka - Vorliegen einer Schwangerschaft, Teilnahme an anderen Studien |
| **Voruntersuchungen:** | Körperliche Untersuchung, orientierende neurologische Anamnese, Befundsichtung |
| **Methoden und Messzeitpunkte:** | **T_0_: Baseline**   - Eignungsbeurteilung (durch den Studienarzt) - Aufklärung der Probanden über die Studie und Einwilligung - Ausgabe Schmerztagebuch   **T_1_: Prä**   - Zweite Eignungsbeurteilung (Schmerztagebuch) - Fragebögen - Messungen der Druckschmerzschwelle - Randomisierung - Schulung entsprechend der Randomisierung   **T_2_: Post** (nach 3 Monaten Behandlung)   - Fragebögen - Messungen der Druckschmerzschwelle - Abschlussbeurteilung, Interview |
| **Interventionen:** | - **Interventionsgruppe:** Schröpfkopfmassage (SKM)   Die Patienten werden in einem Workshop in die Anwendung der Schröpfkopfmassage (durch einen erfahrenen Schröpftherapeuten) eingeführt. Vor der Behandlung wird der Rücken mit einem Massageöl eingerieben. Es werden Schröpfköpfe mit Gummiball auf die intakte Haut gesetzt, ein Vakuum durch das Pumpen mit dem Gummiball erzeugt und der Schröpfkopf dann vorsichtig über die Muskulatur bewegt. Durch das Vakuum und die Bewegung der Haut kommt es zu einer Hyperämisierung und im Verlauf zu einer Entspannung der betreffenden Muskelpartien. Eine Hämatombildung ist in seltenen Fällen möglich, jedoch vorübergehend. Die Patienten wenden die SKM zweimal wöchentlich für drei Monate an.   - **Kontrollgruppe:** Entspannungsübung   Die Patienten werden in einem Workshop in die Anwendung der PMR durch einen Psychologen eingeführt. Sie erhalten dann eine CD mit den jeweiligen Übungen für zuhause, wo sie diese zweimal wöchentlich für drei Monate eigenständig durchführen. |
| **Abhängige Variablen:** | Primärer Zielparameter   - Veränderung der Schmerzintensität (VAS) von T1 und T2   Sekundäre Zielparameter   - Verlauf der Schmerzen und der Medikamenteneinnahme (Tagebuch) - Bewegungsinduzierter Schmerz (PRTM) - Nackenschmerzspezifische Beeinträchtigungen (NDI) - Ängstlichkeit und Depressivität (HADS) - Schmerzqualität (SBL) - Wohlbefinden (FEW16) - Stressfragebogen (PSQ20) - Lebensqualität (SF-36) - Kontrollüberzeugung (GKÜ) - Druckschmerzschwelle (PPT)   Weitere Zielparameter   - Erfahrungen in der Heimanwendung, Sicherheit, Fragen zur Partnerschaft - Exploratorisch: Infekthäufigkeit |
| **Biometrische Auswertung:** | Absolute Veränderungen von T1 (Prä) zu T2 (Post-Treatment) (T2); Multiple Imputation fehlender Werte; Intention-to-treat-Auswertung mittels univariater Kovarianzanalyse (Baseline-Messung und Erwartungshaltung als lineare Kovariaten, Gruppe als klassierte Kovariate) |
| **Leiter der Prüfung:** | Dr. med. Thomas Rampp |
| **Kooperation** | Dr. med. Rainer Stange |
| **Koordination** | Dipl.-Psych. Romy Lauche |
| **Biometrie** | Dipl.-Stat. Rainer Lüdtke |
| **Sponsor:** | - Lehrstuhl für Naturheilkunde und Integrative Medizin - Karl und Veronica Carstens-Stiftung, Am Deimelsberg 36, 45276 Essen, Telefon 0201-56305-0: Projektförderung zur Deckung der Materialkosten (Workshop und Schröpfutensilien) - Weleda AG, Möhlerstraße 3, 73525 Schwäbisch Gmünd, Telefon ++49 7171 919 488: unentgeltliche Bereitstellung der Massageöle |

1. Zusammenfassung

Ziel der Studie ist es, die Wirksamkeit der Schröpfkopfmassage als Heimanwendung im Vergleich zu einer Entspannungstechnik in Heimanwendung bei chronischen Nackenschmerzen zu testen. Zu diesem Zweck sollen bei 84 Patienten mit chronischen Nackenschmerzen die Intensität der Nackenschmerzen, die damit verbundenen Beeinträchtigungen, Lebensqualität und Wohlbefinden sowie die Druckschmerzschwelle getestet werden. Zusätzlich sollen die Infekthäufigkeit als Indikator für das Immunsystem und Erfahrungen in der Anwendung, insbesondere Sicherheit dokumentiert werden.

1. Wissenschaftlicher Hintergrund

Schröpfen ist ein jahrtausendealtes Therapieverfahren, welches in der naturheilkundlichen Behandlung häufig eingesetzt wird. In traditionellen Vorstellungen soll durch den Unterdruck eine Ab- bzw. Ausleitung von Schadstoffen über die Haut erreichen. In moderneren Konzepten handelt es sich um eines von mehreren Verfahren, bei dem es neben einer Hyperämisierung zu verschiedenen neurophysiologischen Veränderungen kommt (Musial, Michalsen, & Dobos, 2008). Zum einen kommt es zu einer Reizung von Mechanorezeptoren im behandelten Segment. Durch diese Stimulation wird die Schmerzinformation an den Neuronen des dorsalen Horns im Rückenmark gehemmt. Zum anderen wird durch die Hyperämisierung die direkte Umgebung der Nozizeptoren verändert, dies führt zu einer Veränderung deren Funktion. Eine schmerzhafte Stimulation außerhalb des Schmerzareals induziert zudem eine diffuse noxious inhibitory control (DNIC), welche durch supraspinale Mechanismen zu einer Schmerzhemmung führen.

Beim Schröpfen werden unterschiedliche Techniken angewendet. Bei einer Schröpfkopfmassage werden Saugglocken aus Glas - so genannte Schröpfköpfe - auf die intakte Haut gesetzt. Durch Absaugen der Luft aus dem Glas entsteht ein Unterdruck, wodurch eine Gewebsfontanelle entsteht, die petechiales Blut und abgesonderte Lymphflüssigkeit enthält. Anschließend wird dieser Vorgang flächig expandiert, indem das Schröpfglas über die meist mit Massageöl vorbehandelte Haut gezogen wird. Diese Methode wird bevorzugt auf den großen Muskelgruppen des Rückens angewendet und führt zu einer gesteigerten Durchblutung und Lockerung des Gewebes, lokal es kann zur Ausprägung eines Hämatoms kommen, welches jedoch vorübergehend ist.

Eine typische Indikation für die Schröpfkopfmassage sind Nackenschmerzen, sofern diese durch Verspannungen der Hals- und Nackenmuskulatur verursacht sind (Abele, 2003; Chirali, 2007). Neben der Schröpfkopfmassage existieren auch andere Methoden wie trockenes Schröpfen mit statischen Saugglocken oder blutiges Schröpfen. Welche Art des Schröpfens indiziert ist, hängt von den jeweiligen Symptomen und der Konstitution des Patienten ab.

Nackenschmerzen sind in der Bevölkerung weit verbreitet. Laut Angaben des Robert Koch-Instituts haben 56 Prozent der Männer und 62 Prozent der Frauen mindestens einmal pro Jahr Rückenschmerzen, 36 Prozent davon leiden unter Beschwerden im Hals- und Schultergürtelbereich. Häufige Ursachen, neben strukturellen Veränderungen bzw. Bandscheibenvorfällen, sind Muskelverspannungen und -verkrampfungen im Schulter- und Nackenbereich, die auf Fehlhaltungen durch falsches und ausdauerndes Sitzen (am Schreibtisch, am PC) (Binder, 2007; Skov, Borg, & Orhede, 1996) aber häufig auch auf seelische Probleme, Angst oder Stress zurückzuführen sind (Linton, 2000). Durch Verkrampfung der Muskulatur werden Durchblutung und Stoffwechsel behindert (Langevin & Sherman, 2007; Larsson, Oberg, & Larsson, 1999) und es kommt neben Schmerzen im betroffenen Areal zu teilweise starken Bewegungseinschränkungen (Steifigkeit). Der Schmerz im Nackenbereich kann zudem in alle Richtungen ausstrahlen und häufig wird eine erhöhte Schmerz- und Berührungssensitivität auch in den umliegenden Arealen beobachtet (La Touche et al., 2010; Scott, Jull, & Sterling, 2005).

Bei vielen Schmerzerkrankungen fällt auf, dass die Patienten keinerlei Beiträge zur Gesundwerdung leisten. Oft wiegen sie sich in dem Glauben, nur eine Behandlung oder Medikamente könnten die Schmerzen in den Griff bekommen. Dabei übersehen sie, dass sie selbst viele Möglichkeiten haben, die Schmerzen positiv zu beeinflussen. Dies ist gerade bei chronischen unspezifischen Nackenschmerzen wichtig, wie die Auswertung der Studie 09-3985 (Blutiges Schröpfen) gezeigt hat. Die Ergebnisse der qualitativen Befragung ergab nämlich, dass die meisten Patienten keine Kontrolle über den Schmerzen zu haben glauben, dies resultiert oft in Hilflosigkeit und Ärger, welche selbst mit erhöhtem Stress einhergehen und damit die Anspannung im Nackenbereich noch verschlimmern (Lauche, Cramer, Haller et al., 2011) Es ist also immens wichtig, den Patienten die Fähigkeiten und Fertigkeiten zu vermitteln, sich auch selbst zu helfen.

Die Schröpfkopfmassage ist eine solche Technik, die sich zunehmend etabliert, so wurde in der Klinik für Naturheilkunde seit Jahren ein Workshop zur Schröpfkopfmassage im Rahmen der Veranstaltungsreihe „Kinderseminar“ angeboten, welcher gut besucht war. Die Schröpfkopfmassage ist leicht zu erlernen und kann jederzeit zuhause angewendet werden. Durch ihre positiven Wirkungen, z.B. die Lockerung der Muskulatur und die Förderung der Durchblutung kann sie auch allgemein zu einem verbesserten Wohlbefinden beitragen. Zudem wird der Schröpfkopfmassage eine gute Wirksamkeit zur Stärkung des Immunsystems nachgesagt.

Erste Ergebnisse aus der Pilotstudie zum Trockenen Schröpfen (Antrag Nr. 09-3986) konnten jedenfalls positive Effekte des Schröpfens auf chronische Nackenschmerzen nachweisen (Lauche, Cramer, Choi et al., 2011)

1. Studienziel

In der hier geplanten Pilotstudie soll nun untersucht werden, welche Wirkungen die Schröpfkopfmassage in einer 3-monatigen Heimanwendung auf chronische Nackenschmerzen hat. Zielparameter sind dabei die Schmerzintensität, damit verbundene Beeinträchtigungen, psychisches Wohlbefinden sowie physiologische Veränderungen der Schmerzverarbeitung.

1. Studiendesign


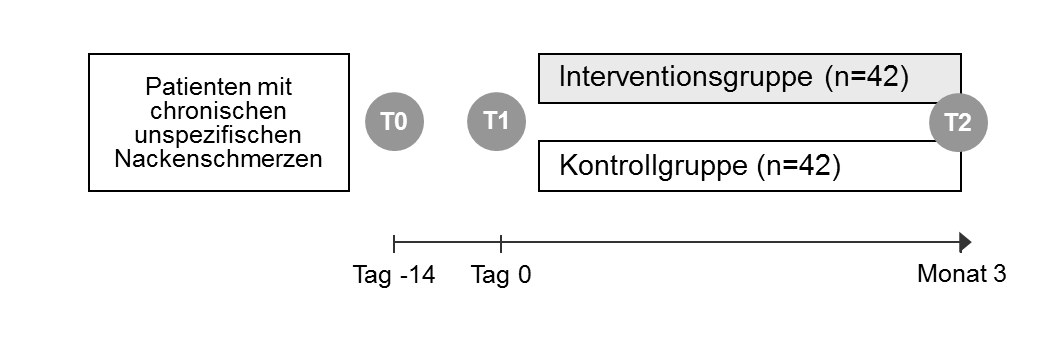


Abbildung 1: Design der Studie.

Die erste medizinische Eignungsuntersuchung findet zu T_0_ statt. Zu T_1_ erfolgt der endgültige Studieneinschluss nach der zweiten Eignungsuntersuchung und Patienten geben ihre Erwartungen bezüglich beider Therapien an. Nach der anschließenden Messung werden die Patienten in eine der beiden Gruppen randomisiert. Die Interventionsgruppe nimmt dann an einem Workshop zur Schröpfkopfmassage teil, die Kontrollgruppe an einer Schulung zur Progressiven Muskelentspannung. Während der Anwendung der jeweiligen Behandlung werden die Patienten telefonisch betreut, um auf etwaige Fragen und Probleme rechtzeitig reagieren zu können, dies dient auch der Kontrolle der Compliance. Nach drei Monaten der Heimanwendung werden die Patienten erneut zu den Messungen zu T_2_ einbestellt. Eine zeitliche Übersicht über die zu erhebenden Parameter ist in Tabelle 1 dargestellt.

1. Patienten
   1. Rekrutierung

Die Patienten werden über Studienaufrufe auf der Webseite der Abteilung und über Presseinserate rekrutiert.

- 1. Einschlusskriterien

Die zu untersuchenden Probanden müssen mindestens 18 Jahre alt sein. Voraussetzung für die Studienteilnahme ist das Vorliegen chronischer Nackenschmerzen seit mindestens 3 Monaten mit einer Intensität von mindestens 45 mm auf der visuellen Analogskala (VAS) (mindestens mittelgradige Schmerzen entsprechend der Klassifikation nach Jensen et al. (Jensen, Chen, & Brugger, 2003)). Es können zudem nur Patienten in die Studie eingeschlossen werden, bei denen ein Partner (Familienangehörige, Freunde, etc.) vorhanden ist, welche/r regelmäßig die erforderlichen Behandlungen durchführen kann.

- 1. Ausschlusskriterien

Eine Studienteilnahme ist nicht möglich bei folgenden Erkrankungen:

- Spezifische Nackenschmerzen aufgrund
  - Entzündlicher muskuloskeletaler Erkrankungen
  - Neurologischer Erkrankungen
  - HWS Prolaps
  - Vorangegangene relevante Traumen (insbesondere Frakturen oder operationsbedingt)
  - Zustand nach Neoplasmen im Bereich der Wirbelsäule
- Angeborene Fehlbildung der Wirbelsäule (Ausnahme: Leichte Skoliose)
- Hauterkrankungen / Entzündungen in den zu behandelnden Arealen / allergische Hautveränderungen
- Hämophilie / Antikoagulation / Thrombozytenaggregationsstörung
- Schwere psychiatrische Erkrankung (z.B. Depression, Suchterkrankung, Schizophrenie)
- Schwere komorbide somatische Erkrankung (z.B. Diabetes mellitus mit bestehender Polyneuropathie, onkologische Erkrankung ohne Remission)
- Langzeitmedikation mit Corticosteroiden > 10mg Prednisolon-Äquivalent, Therapie mit Opiaten
- Vorliegen einer Schwangerschaft
- Teilnahme an anderen klinischen Studien während der Teilnahme (Patienten werden ausdrücklich danach gefragt)

Der Gebrauch von nicht steroidalen Schmerzmedikamenten wird den Patienten während der Studiendauer gestattet. Die Einnahme weiterer Medikamente mit möglichen Einflüssen auf muskuloskeletale Schmerzen, z.B. Muskelrelaxantien, Psychopharmaka usw. wird gestattet, wenn die Medikamente während der Studiendauer dosiskonstant eingenommen werden. Eine in den letzten 6 Wochen vor Studienteilnahme begonnene oder modifizierte Behandlung mit diesen Medikamenten schließt eine Studienteilnahme aus. Die Probanden dokumentieren die Einnahme im Schmerztagebuch.

- 1. Einschlussuntersuchungen
     1. Screening

Die Patienten werden zuerst telefonisch auf Vorliegen der Ein- und Ausschlusskriterien geprüft. Bei Eignung werden sie in die Klinik für Naturheilkunde eingeladen.

- - 1. Erste Eignungsuntersuchung

Zu T_0_ werden eine Anamnese, eine körperliche sowie orientierende neurologische Einschlussuntersuchung durchgeführt. Alle Probanden sind aufgefordert, fachärztliche Befunde beizubringen. Sie erhalten außerdem eine ausführliche Aufklärung über die Studie sowie die Möglichkeit zu einem persönlichen Gespräch mit dem Studienarzt. Vor der Aufnahme in die Studie müssen sie schriftlich ihre Einwilligung zur Studienteilnahme erklären. Auch die Daten derjenigen Patienten, die die Ein- und Ausschlusskriterien nicht erfüllen oder ihre Einwilligung verweigern, werden dokumentiert.

- - 1. Zweite Eignungsuntersuchung

Nach der ersten Eignungsuntersuchung wird jedem Patienten ein Schmerztagebuch für 14 Tage ausgehändigt, in welches er einmal täglich jeweils abends seine Schmerzen eintragen soll. Zu T_1_ erfolgt die Kontrolle des Schmerztagebuchs. Nur Patienten, die das Schmerztagebuch regelmäßig geführt haben (mindestens an 80% der Tage) und deren Schmerzen im Durchschnitt 45mm auf der VAS betragen, werden für die Studie randomisiert und weiterbeobachtet.

- 1. Randomisierung

Nach Abschluss der ersten Messung und vor Therapiebeginn werden die Patienten über eine nicht-stratifizierte Blockrandomisation mit zufällig variierender Blocklänge einer der beiden Behandlungsgruppen zugeordnet. Hierzu werden vom Biometriker über den Zufallszahlengenerator „ranuni“ der SAS®-Software (release 9.2, SAS Inc., Cary NC, USA) Zufallszahlen gezogen, auf deren Basis er versiegelte, undurchsichtige und aufsteigend nummerierte Briefumschläge erstellt. Falls ein Patient die Ein- und Ausschlusskriterien erfüllt, wird der Briefumschlag mit der niedrigsten Patientennummer geöffnet und der dort verzeichneten Behandlung zugeordnet.

1. Intervention

Jede Behandlung wird von den Patienten über 3 Monate zuhause selbst durchgeführt. Dazu erhalten die Patienten eine ausführliche Einweisung, schriftliches Informationsmaterial und das notwendige Material. Die Patienten der Kontrollgruppe erhalten eine Einweisung und eine Entspannungs-CD mit nach Hause. Bei Fragen stehen jederzeit die Studienkoordinatorin bzw. der Studienarzt zur Verfügung.

- 1. Schröpfkopfmassage

Die Patienten und deren Partner werden in einem Workshop in die Anwendung der Schröpfkopfmassage eingewiesen. Der Workshop, der von einer erfahrenen Schröpftherapeutin geleitet und von einer Ärztin und einer Psychologin begleitet wird, behandelt u.a. die Geschichte und die Hintergründe der Schröpfkopfmassage. Es werden Indikationen, Kontraindikationen sowie die grundlegende Technik vorgestellt. Im Anschluss erhalten alle Teilnehmer ein Schröpfglas mit Gummiball sowie eine Flasche Massageöl (200ml, Weleda Arnika Massageöl) und die Technik wird unter Aufsicht ausprobiert und eingeübt. Insbesondere wird dabei auf das individuelle Beschwerdebild der jeweiligen Patienten eingegangen. Nach dem Workshop dürfen die Patienten das Glas, das Massageöl sowie eine schriftliche Anleitung mit nach Hause nehmen. Während der gesamten Behandlungsphase werden die Patienten telefonisch betreut, dies soll Studienabbrüche vermeiden helfen und die Compliance verbessern.

Die Schröpfkopfmassage selbst wird auf der intakten Haut praktiziert. Zur Vorbereitung wird der Rücken des Patienten mit Arnika-Massageöl von Weleda eingerieben, ein Schröpfkopf mit Gummiball aufgesetzt und mithilfe des Gummiballs ein Unterdruck erzeugt. Der Schröpfkopf wird dann auf beiden Körperseiten in langen Zügen langsam über die Paravertebralmuskulatur vom Occiput bis in Höhe der mittleren Brustwirbelsäule sowie über den oberen Rand des Musculus trapezius gezogen. Die Schmerzareale werden dabei besonders intensiv behandelt. Entlang dieser Züge können sich Petechien bilden. Eine Schröpfkopfmassage dauert insgesamt 10 Minuten, nach der Massage wird eine Ruhephase empfohlen. Im Rahmen der Studie wenden die Patienten die Schröpfkopfmassage zweimal wöchentlich zwischen 16 und 20 Uhr für 3 Monate an.

- 1. Entspannungsübung

Die Patienten werden in einem Workshop in die Anwendung der Progressiven Muskelentspannung (PME) eingeführt. Eine Diplom-Psychologin stellt den Patienten in einem Workshop die Geschichte, die Hintergründe und den Ablauf der PME dar, anschließend wird eine PME mit den Patienten durchgeführt. Anschließend werden Erfahrungen ausgetauscht und alle offenen Fragen beantwortet. Am Ende des Workshops erhalten die Patienten eine CD (Techniker Krankenkasse) mit nach Hause und wenden die Entspannungsübung zweimal wöchentlich zwischen 16 und 20 Uhr für 3 Monate an. Während der Behandlungsphase werden die Patienten telefonisch betreut, dies dient der Vermeidung von Studienabbrüchen sowie der Kontrolle der Compliance. Im Anschluss an die Studie erhalten diese Patienten ebenfalls die Möglichkeit, an einem Workshop zur Schröpfkopfmassage teilzunehmen. Zudem werden auch ihnen das Schröpfglas sowie das Massageöl kostenfrei zur Verfügung gestellt.

1. Methoden

Eine Übersicht über die jeweiligen Messmethoden zu den einzelnen Zeitpunkten ist in Tabelle 1 dargestellt.

| **Parameter** | **T0** | **T1** | **T2** |
| --- | --- | --- | --- |
| Anamnese, Soziodemographische Daten | X |  |  |
| Erwartungshaltung |  | X |  |
| Fragebögen:  Nackenschmerzen (VAS), Schmerzen in Bewegung (PRTM), Schmerzqualität (SBL) Beeinträchtigung (NDI), Wohlbefinden (FEW16), Ängstlichkeit und Depressivität (HADS), Stresserleben (PSQ20), Lebensqualität (SF-36), Kontrollüberzeugung (GKÜ) |  | X | X |
| Druckschmerzempfindlichkeit (PPT) |  | X | X |
| Schmerztagebuch (Medikamente und VAS) | X | X | X |
| Evaluation, Sicherheit |  |  | X |
| Exploratorische Fragen:  Veränderungen der Partnerschaft, Infekthäufigkeit | X | X | X |

Tabelle 1: Übersicht über die Erhebung von Fragebögen und Messungen zu den jeweiligen Messzeitpunkten.

- 1. Messmethoden

Zum einen werden patienten­spezifische Daten zur Sozialdemografie (Alter, Geschlecht, Größe, Gewicht, sozioökonomischer Sta­tus), der Erkran­kungs­dauer, bisherigen Thera­pien und zur Medikamen­ten­einnahme erhoben.

Mittels Schmerztagebuch, in das die Patien­ten täglich ihre Schmerzstärke auf einer 100mm-VAS eintragen, wird der Verlauf der Schmerzen, die Einnahme von Medikamenten sowie begleitende Behandlungen während der Behandlungsphase verfolgt.

Weiterhin werden folgende standardisierte Fragebogeninstrumente verwendet:

- Fragen zur Schmerzintensität in Ruhe (**VAS, 0-100mm**)
- Schmerz bei Bewegung (**PRTM**) (Irnich et al., 2001). Mittels PRTM wird der bewegungsinduzierte Schmerz erfasst, der durch die Bewegung des Kopfes in die 6 Bewegungsrichtungen entsteht. Die Einschätzung erfolgt auf einer VAS (0-100mm).
- Beeinträchtigungen (**NDI**) (Vernon & Mior, 1991). Mittels 10 Fragen werden die Auswirkungen der Nackenschmerzen auf Tätigkeiten des Alltags eingeschätzt.
- Schmerzqualität (**SBL**) (Korb & Pfingsten, 2003). Diese 12-Item Kurzversion der Schmerzempfindungsskala erlaubt die Messung und differenzierte Beschreibung der subjektiv wahrgenommenen Schmerzen auf sensorischen und affektiven Dimensionen.
- Ängstlichkeit und Depressivität (**HADS**) (Herrmann, Buss, & Snaith, 1995).
- Lebens­qualität (**SF-36**) (Bullinger & Kirchberger, 1998). Mit dem SF-36 wird die gesundheitsbezogene Lebensqualität auf insgesamt 8 Skalen und 2 Gesamtskalen (psychisch, physisch) beurteilt. Für den SF-36 liegen umfangreiche Normwerte vor.
- Gesundheitsbezogene Kontrollüberzeugungen (**GKÜ**) (Hasenbring, 1989). Mit diesem Fragebogen wird die Vorstellung darüber, welchen Personen oder Umstände Einfluss auf den Krankheitsverlauf nehmen, erfasst.
- Wohlbefinden (**FEW16**) (Kolip & Schmidt, 1999). Dieser Fragebogen erfasst vier Dimensionen habituellen körperlichen Wohlbefindens Erwachsender mit 16 Fragen.
- Stressempfinden (**PSQ20**) (Fliege et al., 2005). Mit diesem Fragebogen wird der wahrgenommene Stress unabhängig von spezifischen Ereignissen auf vier Dimensionen erfasst.

Die Druckschmerzschwelle wird mittels Algometer der Firma Somedic erfasst. Diese Methode wurde bereits in den Studien 09-3895, 09-3896, 09-3987 angewendet und im Ethikprotokoll beschrieben. Der Druck mit mittels drei kontinuierlicher Rampen aufsteigender Intensität (40 kPa/s) appliziert, bis der Patient mittels Knopfdruck eine Veränderung der Wahrnehmung von ausschließlich Druck hin zu schmerz­haftem Druck angibt. Der zu diesem Zeitpunkt anliegende Druck wird als Erreichen der Schmerzschwelle protokolliert und nach 3 Durchgängen gemittelt. Die Druckschmerzschwelle wird am individuellen Schmerzmaximum sowie bilateral an drei anatomisch definierten Punkten (M. levator scapu­lae, M. semispinalis capitis, M. trapezius) sowie an der rechten Handinnenfläche (über dem Thenar) zu Demonstrationszwecken und zur Reliabilitätsbestimmung erhoben (Johnston, Jimmieson, Jull, & Souvlis, 2008).

Im Anschluss an die Intervention werden die Erfahrungen mit der Anwendung, zur Compliance und zu beobachteten Veränderungen mittels Interview erfasst. Hier soll unter anderen auch die Sicherheit des Verfahrens beurteilt werden.

Explorativ wird zudem der Einfluss der Behandlung auf die Partnerschaft erfasst. Grundlage dafür bildet das halbstandardisierte Interview, welches verschiedene Aspekte der Partnerschaft erfragt. Zuletzt soll die Häufigkeit von Infekten eingeschätzt werden. Dies ist insbesondere relevant, da die Studie in den Wintermonaten durchgeführt werden soll. Dies kann als Grundlage für weitere Studien dienen.

- 1. Messzeitpunkte

Die Zielparameter werden an den folgenden Messzeitpunkten erhoben: Unmittelbar vor der Intervention (T_1_) sowie direkt nach Therapieende (T_2_), siehe Design. Auf eine Katamnese wird verzichtet, da die Patienten die Methode der Schröpfkopfmassage, sofern sie ihnen Linderung verschafft hat, vermutlich weiter anwenden werden und dadurch die Ergebnisse verfälschen. Zudem erscheint es für die Patienten der Kontrollgruppe nicht zumutbar, weitere 3 Monate auf den Workshop zu warten.

Das Schmerztagebuch wird während der gesamten Studie geführt. Das halbstrukturierte Interview findet direkt nach Therapieende zu T_2_ statt.

1. Zielparameter
   1. Primäre Zielgröße

Als primärer Zielparameter wird die Veränderung der Schmerzintensität (VAS) von T1 und T2 definiert.

- 1. Sekundäre Zielgrößen

Als sekundäre Zielparameter werden die folgenden Variablen definiert:

- Veränderung der Schmerzintensität und Medikamentengebrauch mittels Tagebuch
- Bewegungsinduzierter Schmerz (PRTM)
- Nackenschmerzspezifischen Beeinträchtigungen (NDI)
- Schmerzqualität (SBL)
- Wohlbefinden (FEW16)
- Ängstlichkeit und Depressivität (HADS)
- Stressempfinden (PSQ20)
- Lebensqualität (SF-36)
- gesundheitsbezogenen Kontrollüberzeugung (GKÜ)
- Druckschmerzschwelle (PPT)
  1. Weitere Studienparameter

Die Erfahrungen der Patienten während der Anwendung, die Compliance sowie beobachtete Veränderungen werden rein deskriptiv erfasst. Ebenso werden Sicherheitsaspekte deskriptiv dokumentiert.

Der Einfluss der Behandlung auf die Partnerschaft wird mittels qualitativem Interview erhoben. Die Auswertung erfolgt mittels qualitativer Inhaltsanalyse nach Mayring (Mayring, 2008).

1. Statistik
   1. Fallzahlschätzung

Die im Vorfeld des Planungsprozesses durchgeführte Literaturrecherche ergab keine klinische Interventionsstudie, aus der sich eine Aussage über die Größe des klinisch relevanten Effekts einer Schröpfkopfmassage bei chronischen Nackenschmerzen ableiten ließe. Zur statistischen Fallzahlplanung wurde die Studie 09-3987 zur Schröpfkopfmassage bei chronischen Nackenschmerzen herangezogen. Die Auswertung der Daten ergab eine signifikante Gruppendifferenz (ANCOVA) nach Behandlung von -14,3 mm auf der VAS. Die geschätzte Effektstärke betrug Cohens d=0.66, welches einem mittleren bis hohen Effekt entspricht. Diese Effektgröße wurde für die Fallzahlschätzung zugrunde gelegt, wohlwissen, dass bei der früheren Studie die Behandlung in der Klinik von einem Fachmann durchgeführt wurde. Durch das veränderte Setting sowie die fehlende Zuwendung durch den Therapeuten erscheint es plausibel, dass die unspezifischen Effekte geringer ausfallen könnten. Andererseits soll die Behandlung nun von einem empathischen Partner durchgeführt werden, diese persönliche Zuwendung sollte diesen Effekt letztlich wieder ausgleichen können. Zudem sind in der vorliegenden Studie mehr Behandlungen geplant, so dass der zu erwartende Effekt eher noch größer ist und die Zahl der benötigten Patienten eher überschätzt wird. Insgesamt ist es jedoch schwierig, die Einflüsse dieser Effekte a priori abzuschätzen.

Um einen Gruppenunterschied von d=0.66 zu einem Niveau α=0.05 mit einer Power von 80% nachweisen zu können, benötigt ein zweiseitiger t-Test eine Fallzahl von insgesamt 76 Patienten (38 je Gruppe). Um eventuelle Powerverluste durch eine Drop-Out-Rate von 10% auszugleichen, sollen insgesamt 84 Patienten eingeschlossen werden (42 je Gruppe).

- 1. Statistische Auswertung

Alle Auswertungen, insbesondere die Auswertung des Hauptzielkriteriums, erfolgen auf der Basis der intention-to-treat-Population, d.h. aller randomisierten Patienten. Fehlende Werte werden diese nach dem Prinzip der multiplen Imputationen mittels der MCMC-Methode mehrfach ersetzt. Insgesamt werden so 50 verschiedene, vollständige Datensätzen erzeugt (Prozedur PROC MI der SAS/STAT®-Software), einzeln analysiert und die jeweils ermittelten Gruppenunterschiede geeignet zusammengefasst (Prozedur PROC MIANALYZE).

An das Hauptzielkriterium wird ein Kovarianzanalysemodell angelegt, in dem die Gruppenzugehörigkeit (binäre Kovariate), der Baseline-Werte (lineare Kovariable) und die Erwartungshaltung (lineare Kovariate) als unabhängige fixe Faktoren modelliert werden. Innerhalb dieses Modells wird der adjustierte Gruppenunterschied (inclusive 95%-Konfidenzintervall) geschätzt und mittels eines zweiseitigen t-Tests zum Niveau α=0.05 auf Überlegenheit der Schröpfkopfmassage getestet.

Eine Zwischenauswertung findet nicht statt. Eine multiple Adjustierung der Ergebnisse ist daher nicht notwendig.

Für die Nebenzielkriterien werden Kovarianzmodelle verwendet, die denen des Hauptzielkriteriums entsprechen. Die berichteten Konfidenzintervalle und p-Werte für die ajdustierten Gruppenunterschiede werden noch nur deskriptiv interpretiert.

1. Nutzen/Risiko-Abwägung
   1. Schröpfkopfmassage

Die Schröpfkopfmassage gilt im Allgemeinen als sehr sicher und wird in der Klinik für Naturheilkunde und Integrative Medizin routinemäßig als Therapie bei unterschiedlichsten Schmerzen angewendet. Seit mehreren Jahren werden Patienten in der SKM erfolgreich und mit positivem Feedback unterrichtet. Bei richtiger Indikationsstellung sind außer bei einer ausgeprägten Altershaut oder Hautentzündungen keine Kontraindikationen bekannt. Eine mögliche Hämatombildung ist vorübergehend.

- 1. Entspannungsübung

Entspannungsübungen werden bei verschiedenen Erkrankungen, insbesondere bei stressassoziierten Erkrankungen erfolgreich angewendet. Die Wirkung ist in den meisten Fällen unspezifisch. Es sind keine Nebenwirkungen bekannt.

- 1. Untersuchungsmethoden

Von den Fragebögen sind keine Risiken bekannt. Die Messung der Druckschmerzschwelle erfolgt mittels einer etablierten Methode, die nur unter sehr pathologischen Bedingungen zu einer verstärkten Wahrnehmung von Schmerzen führt. Im Falle der Ermittlung der Druckschmerzschwelle wird die Messung unmittelbar bei Erreichen der Druckschmerzschwelle abgebrochen (der Proband betätigt hierzu einen Signalknopf), damit ist das Risiko für die Patienten als sehr gering einzuschätzen.

- 1. Zusammenfassende Nutzen/Risiko-Abwägung

In der Klinik für Naturheilkunde und Integrative Medizin der Kliniken Essen-Mitte wird die Schröpfkopfmassage schon seit längerer Zeit mit sehr gutem Erfolg bei Schmerzen des Bewegungsapparates angewandt, eine erste noch nicht veröffentlichte Pilotstudie (09-3987) zeigt vielversprechende Ergebnisse. Angesichts der langjährigen Erfahrung mit dieser Therapiemethode und der einfachen Handhabbarkeit ist von einem insgesamt günstigen Nutzen-Risiko-Verhältnis auszugehen.

1. Studienprotokollerstellung und Ethikantrag

Der Leiter der klinischen Prüfung holt das Votum bei der zuständigen Ethikkommission ein. Die Studie wird erst nach positivem Votum der Ethikkommission der Universität Essen begonnen. Die Ethikkommission muss bei Protokollabweichungen, schwerwiegenden oder unbekannten unerwünschten Ereignissen oder neuen Erkenntnissen, die auf eine Gefährdung der Probandensicherheit hindeuten, informiert werden.

1. Patientenversicherung

Eine verschuldensunabhängige Patientenversicherung wird für diese Studie nicht abgeschlossen. Ebenso besteht keine Wegeunfallversicherung. Hierauf werden die Patienten hingewiesen.

1. Patienteninformation, Einwilligung und Datenschutz

Nach § 4(1) BDSG (Bundesdatenschutzgesetz) ist die Erhebung, Verarbeitung und Nutzung personenbezogener Daten nur zulässig, wenn der Betroffene eingewilligt hat. Zum Zwecke der Anonymisierung der Daten wird auf allen Fragebögen und Anamnesebögen lediglich ein Zahlencode verwendet.

Die teilnehmende Person wird durch einen Prüfer über Wesen, Bedeutung, Risiken und Tragweite der klinischen Prüfung sowie über ihr Recht, die Teilnahme an der klinischen Prüfung zu beenden aufgeklärt. Es wird eine allgemeinverständliche Patienteninformation ausgehändigt und es wird Gelegenheit zu einem Beratungsgespräch mit einem Prüfer über die sonstigen Bedingungen der Durchführung der Studie gegeben. Eine Einwilligung zur Studienteilnahme kann jederzeit widerrufen werden, ohne dass der betroffenen Person dadurch Nachteile entstehen. In Fall des Widerrufs der Einwilligung werden alle erhobenen Daten der Person gelöscht.

Die betroffene Person ist über Zweck und Umfang der Erhebung und Verwendung personenbezogener Daten, insbesondere von Gesundheitsdaten zu informieren. Betroffene Personen, die dieser Weitergabe nicht zustimmen, werden nicht in die klinische Prüfung eingeschlossen. Die betroffene Person wird darüber informiert, dass die erhobenen Daten soweit erforderlich

a) zur Einsichtnahme zwecks Überprüfung der ordnungsgemäßen Durchführung der klinischen Prüfung bereitgehalten werden,

b) pseudonymisiert an den wissenschaftlichen Leiter und das biometrische Institut zum Zwecke der wissenschaftlichen Auswertung weitergegeben werden.

c) und nur in anonymisierter Form wissenschaftlich veröffentlicht werden.

1. Finanzierung und wirtschaftliche Interessen

Die Durchführung der Studie wird mit Mitteln des Lehrstuhls für Naturheilkunde finanziert. Weiterhin wird die Studiendurchführung mit einer Projektförderung der gemeinnützigen Karl und Veronica Carstens-Stiftung (Karl und Veronica Carstens-Stiftung, Am Deimelsberg 36, 45276 Essen, Telefon 0201-56305-0, www.carstens-stiftung.de) unterstützt. Diese Förderung umfasst die Materialkosten, d.h. die Kosten für die Workshops sowie die Schröpfutensilien, welche den Patienten kostenfrei zur Verfügung gestellt werden. Die WELEDA AG (Weleda AG, Möhlerstraße 3, 73525 Schwäbisch Gmünd, Telefon ++49 7171 919 488, Telefax ++49 7171 919 87 488, http://www.weleda.de) stellt unentgeltlich die Massageöle für die Patienten zur Verfügung. Sowohl die Karl und Veronica Carstens-Stiftung als auch die WELEDA AG haben zu keinem Zeitpunkt Einfluss auf die Studie.

1. Studienabbruch

Die Studie wird regulär nach der Rekrutierung von 84 Probanden abgeschlossen. Sie kann unter den nachfolgenden Bedingungen vorzeitig abgebrochen werden, wenn:

- die erforderlichen Rekrutierungszahlen nicht innerhalb von 6 Monaten erreicht werden
- schwerwiegende Protokollverletzungen auftreten
- die Dokumentationsbögen mangelhaft oder vorsätzlich falsch ausgefüllt werden
- gesetzliche oder ethische Bestimmungen werden nicht eingehalten

Ein Abbruch aus den o.g. Gründen ist nur in Übereinstimmung zwischen Studienleiter und den Prüfärzten möglich. Häufen sich schwerwiegende unerwünschte Effekte oder Nebenwirkungen, so kann der Studienleiter in alleiniger Entscheidung die Studie abbrechen.

Die Prüfung endet für den einzelnen Patienten mit Erreichen des im Prüfplan vorgesehenen Zeitpunktes für die Abschlussuntersuchung oder mit dem Widerruf der Einwilligung. Eine Beendigung der Behandlung bei einem Patienten kann ferner durch folgende Gründe bedingt sein:

- Auftreten schwerwiegender Ereignisse
- Auftreten schwerer interkurrenter Erkrankungen
- Nichtbefolgen der ärztlichen Anweisung, wenn dadurch ein nicht vertretbares Risiko für den Patienten besteht.

1. Eignung der Prüfstelle und wissenschaftlichen Einrichtung

Die Einrichtung verfügt über mehrjährige Erfahrung in der Durchführung klinischer Studien. Es stehen in Studien erfahrenes Personal, angemessene Räumlichkeiten und durchgehend nutzbare PC-Arbeitsplätze zur Verfügung.

1. Literatur

Abele, J. (2003). *Das Schröpfen: eine bewährte alternative Heilmethode* (5. ed.). München: Urban & Fischer Verlag.

Binder, A. (2007). The diagnosis and treatment of nonspecific neck pain and whiplash. *Europa Medicophysica, 43*(1), 79-89.

Bullinger, M., & Kirchberger, I. (1998). *SF-36. Fragebogen zum Gesundheitszustand. Handanweisung*. Göttingen: Hogrefe.

Chirali, I. (2007). *Traditional chinese medicine cupping therapy* (2nd ed.). Philadelphia, PA: Elsevier Churchill Livingston.

Fliege, H., Rose, M., Arck, P., Walter, O. B., Kocalevent, R. D., Weber, C., et al. (2005). The perceived stress questionnaire (PSQ) reconsidered: Validation and reference values from different clinical and healthy adult samples. *Psychosomatic Medicine, 67*(1), 78-88.

Hasenbring, M. (1989).
Laienhafte ursachenvorstellungen und erwartungen zur Beeinflußbarkeit einer krebserkrankung - erste ergebnisse einer studie an krebspatienten. In C. Bischoff, & H. Zenz (Eds.), *Patientenkonzepte von körper und krankheit* (pp. 25-38). Bern: Huber.

Herrmann, C., Buss, U., & Snaith, R. P. (1995). *Hospital Anxiety and Depression Scale - Deutsche Version (HADS-D). Manual*. Bern: Hans Huber.

Irnich, D., Behrens, N., Molzen, H., Konig, A., Gleditsch, J., Krauss, M., et al. (2001, Jun 30). Randomised trial of acupuncture compared with conventional massage and "sham" laser acupuncture for treatment of chronic neck pain. *BMJ (Clinical Research Ed.), 322*, 1574-1578.

Jensen, M. P., Chen, C., & Brugger, A. M. (2003). Interpretation of visual analog scale ratings and change scores: A reanalysis of two clinical trials of postoperative pain. *The Journal of Pain : Official Journal of the American Pain Society, 4*(7), 407-414.

Johnston, V., Jimmieson, N. L., Jull, G., & Souvlis, T. (2008). Quantitative sensory measures distinguish office workers with varying levels of neck pain and disability. *Pain, 137*(2), 257-265.

Kolip, P., & Schmidt, B. (1999). Der Fragebogen zur Erfassung körperlichen Wohlbefindens (FEW16). Konstruktion und erste Validierung. *Zeitschrift für Gesundheitspsychologie, 7*, 77-87.

Korb, J., & Pfingsten, M. (2003). Der deutsche schmerzfragebogen - implementierte psychometrie. *Schmerz, 17*, S47.

La Touche, R., Fernandez-de-Las-Penas, C., Fernandez-Carnero, J., Diaz-Parreno, S., Paris-Alemany, A., & Arendt-Nielsen, L. (2010). Bilateral mechanical-pain sensitivity over the trigeminal region in patients with chronic mechanical neck pain. *The Journal of Pain : Official Journal of the American Pain Society, 11*(3), 256-263.

Langevin, H. M., & Sherman, K. J. (2007). Pathophysiological model for chronic low back pain integrating connective tissue and nervous system mechanisms. *Medical Hypotheses, 68*(1), 74-80.

Larsson, R., Oberg, P. A., & Larsson, S. E. (1999). Changes of trapezius muscle blood flow and electromyography in chronic neck pain due to trapezius myalgia. *Pain, 79*(1), 45-50.

Lauche, R., Cramer, H., Choi, K. E., Rampp, T., Saha, F. J., Dobos, G. J., et al. (2011). The influence of a series of five dry cupping treatments on pain and mechanical thresholds in patients with chronic non-specific neck pain - a randomised controlled pilot study. *BMC Complementary and Alternative Medicine, 11*(1), 63.

Lauche, R., Cramer, H., Haller, H., Musial, F., Langhorst, J., Dobos, G. J., et al. (2011). “My body shrinks” – the influence of traditional cupping on the body image in patients with chronic non-specific neck pain. *Journal of Traditional Chinese Medicine, 31*(Supplement), 46-46.

Linton, S. J. (2000). A review of psychological risk factors in back and neck pain. *Spine, 25*(9), 1148-1156.

Mayring, P. (2008). *Qualitative Inhaltsanalyse. Grundlagen und Techniken* (10. Auflage ed.). Weinheim und Basel: Beltz.

Musial, F., Michalsen, A., & Dobos, G. (2008). Functional chronic pain syndromes and naturopathic treatments: Neurobiological foundations. *Forschende Komplementarmedizin (2006), 15*(2), 97-103.

Scott, D., Jull, G., & Sterling, M. (2005). Widespread sensory hypersensitivity is a feature of chronic whiplash-associated disorder but not chronic idiopathic neck pain. *The Clinical Journal of Pain, 21*(2), 175-181.

Skov, T., Borg, V., & Orhede, E. (1996). Psychosocial and physical risk factors for musculoskeletal disorders of the neck, shoulders, and lower back in salespeople. *Occupational and Environmental Medicine, 53*(5), 351-356.

Vernon, H., & Mior, S. (1991). The neck disability index: A study of reliability and validity. *Journal of Manipulative and Physiological Therapeutics, 14*(7), 409-415.

1. Anlagen

Patienteninformation, Einwilligungserklärung

Schmerztagebuch, Fragebögen, Informationsmaterial für die Patienten

Aufruf auf der Webseite, Presseinserat
