# Supplementary material for: Effectiveness of Home-Based Cupping Massage Compared to Progressive Muscle Relaxation in Patients with Chronic Neck Pain—A Randomized Controlled Trial
Source: PLoS One. 2013 Jun 7;8(6):e65378. doi: 10.1371/journal.pone.0065378 (PMC3676414; doi:10.1371/journal.pone.0065378)
Supplement: Protocol S2 — Trial protocol. English translation of the study protocol submitted to the ethics committee of the University Hospital Essen. (DOCX) [file pone.0065378.s002.docx]

Study protocol for submission to the Ethics Commission of the University of Duisburg-Essen

**Project title:**

Randomised controlled trial on the effectiveness of home based cupping massage in for chronic neck pain

University of Duisburg-Essen

Chair of Complementary and Integrative Medicine of the Alfried Krupp von Bohlen und Halbach foundation

Essen-Mitte Clinics

Department of Internal and Integrative Medicine

Knappschaftskrankenhaus

Am Deimelsberg 34a

45276 Essen

**Clinic director**

Prof. Dr. med. Gustav Dobos

Kliniken Essen-Mitte

Am Deimelsberg 34a

45276 Essen

Tel.: 0201 - 174 25001

Fax: 0201 - 174 25000

**Principle investigator and study physician**

Dr. med. Thomas Rampp

Kliniken Essen-Mitte

Am Deimelsberg 34a

45276 Essen

Tel.: 0201 - 174 25013

Fax: 0201 - 174 25000

**Scientific cooperation**

Dr. med. Rainer Stange

Immanuel Krankenhaus Berlin (Standort Berlin-Wannsee)

Königstraße 63

D-14109 Berlin

Tel.: 030 - 80505 - 690

Fax: 030 - 80505 - 288

**Study coordinator**

Dipl.-Psych. Romy Lauche

Kliniken Essen-Mitte

Am Deimelsberg 34a

45276 Essen

Tel.: 0201 - 174 25054

Fax: 0201 - 174 25000

**Statistics**

Dipl.-Stat. Rainer Lüdtke

Karl und Veronica Carstens-Stiftung

Am Deimelsberg 36

45276 Essen

Tel.: 0201 - 5630516

Fax: 0201 – 563050

By signing below I certify that I have read the proposed protocol described above and I agree to the terms. I certify that all questions arising from the protocol have been answered sufficiently and I assure that I will conduct the clinical trial according to the protocol.

**Clinic director**

_________________________________ _________________________________

Place, Date Prof. Dr. med. Gustav Dobos

**Prinicple investigator**

_________________________________ _________________________________

Place, Date Dr. med. Thomas Rampp

**Scientific cooperation**

_________________________________ _________________________________

Place, Date Dr. med. Rainer Stange

**Study coordinator**

_________________________________ _________________________________

Place, Date Dipl.-Psych. Romy Lauche

**Statistician**

_________________________________ _________________________________

Place, Date Dipl.-Stat. Rainer Lüdtke

Gliederung

[1. Synopsis 5](#_Toc343090060)

[2. Summary 8](#_Toc343090061)

[3. Background 8](#_Toc343090062)

[4. Aim of the study 9](#_Toc343090063)

[5. Study design 9](#_Toc343090064)

[6. Patients 10](#_Toc343090065)

[6.1. Recruitment 10](#_Toc343090066)

[6.2. Inclusion criteria 10](#_Toc343090067)

[6.3. Exclusion criteria 10](#_Toc343090068)

[6.4. Examination 11](#_Toc343090069)

[6.5. Randomization 11](#_Toc343090070)

[7. Intervention 11](#_Toc343090071)

[7.1. Cupping massage 12](#_Toc343090072)

[7.2. Relaxation training 12](#_Toc343090073)

[8. Methods 12](#_Toc343090074)

[8.1. Measurement instruments 13](#_Toc343090075)

[8.2. Measurement time points 14](#_Toc343090076)

[9. Outcomes 14](#_Toc343090077)

[9.1. Primary outcome 14](#_Toc343090078)

[9.1. Secondary outcome 14](#_Toc343090079)

[9.2. Exploratory outcomes 14](#_Toc343090080)

[10. Statistics 15](#_Toc343090081)

[10.1. Estimation of required sample size 15](#_Toc343090082)

[10.2. Statistical analysis 15](#_Toc343090083)

[11. Risk Benefit Analysis 15](#_Toc343090084)

[11.1. Cupping massage 15](#_Toc343090085)

[11.2. Relaxation 16](#_Toc343090086)

[11.3. Measurements 16](#_Toc343090087)

[11.4. Summary of Risks and benefits 16](#_Toc343090088)

[12. Study protocol 16](#_Toc343090089)

[13. Insurance 16](#_Toc343090090)

[14. Patient information, informed consent and data protection 16](#_Toc343090091)

[15. Financing and conflict of interest 17](#_Toc343090092)

[16. Termination of study 17](#_Toc343090093)

[17. Eligibility of trial site 18](#_Toc343090094)

[18. References 18](#_Toc343090095)

1. Synopsis

| **Acronym** | NaSK |
| --- | --- |
| **Title:** | Randomised controlled trial on the effectiveness of home based cupping massage in for chronic neck pain |
| **Background and Aim of the study:** | In this study, the effectiveness of home- based cupping massage shall be tested in comparison to relaxation training in chronic non-specific neck pain patients. Therefore neck pain intensity (VAS), pain at motion (PRTM), pain quality (SBL), specific disability (NDI), well-being (FEW16), stress perception (PSQ20) quality of life (SF-36), anxiety and depression (HADS) as well as locus of control (GKÜ) will be measured. Pressure pain sensitivity (PPT) will be determined at pre-specified muscles.  In a daily log, pain intensity, medication and concurrent therapies will be noted. Personal experiences, changes and the influence of the therapy on partnership issues will be investigated with a qualitative interview at the end of the study.  Safety will also be evaluated. |
| **Design:** | Randomised controlled monocentric interventional study with 2 parallel groups:   - Home-based cupping massage, introduction in workshop, afterwards 2xweekly application for 12 weeks - Progressive muscle relaxation, introduction in workshop, afterwards 2xweekly application for 12 weeks. After termination of the study patients in this group will be offered home-based cupping massage.   Outcome assessors are blinded. |
| **Study centre:** | Essen-Mitte Clinics, Department of Internal and Integrative Medicine |
| **Number of patients:** | 42 Patients per group, i.e. 84 Patients in 2 groups. Estimation of patients needed according to ethics protocol no. 09-3987. |
| **Duration of study:** | Per Patient 4 months, 6 months for the complete study |
| **Inclusion criteria:** | - Age between 18 and 75 years - chronic non-specific neck pain for at least 3 months with intensity > 45mm VAS - Partner for administration of cupping massage (family, friends) - Medical records for anamnesis |
| **Exclusion criteria:** | - Specific causes for neck pain, such as : - Inflammatory diseases - Neurological disorders - Disc prolapse - Trauma, e.g. whiplash, fractures, operation at the upper spine) - Neoplasms of the spine - Congenital spine malformation - Skin disease/inflammation/allergic reactions at the areas to be treated - Haemophilia, anticoagulant medication - Severe psychiatric disorder - Severe comorbid disorder (e.g. diabetes mellitus with neuropathy, oncological disease) - Long-time medication with corticosteroids >10mg Prednisolon-equivalent - Opiate medication - Recently started or modified medication with influences on musculoskeletal pain, such as muscle relaxants, psychopharmacological - Pregnancy - Current participation in other clinical studies |
| **Examination:** | Physical examination, orienting neurological anamnesis, check of medical records |
| **Methods:** | **T_0_: Baseline**   - Examination (study physician) - Obtaining written informed consent of patients - Daily log   **T_1_: Prä**   - Second examination (daily log) - questionnaires - pressure pain thresholds - randomization - workshops   **T_2_: Post** (after 12 weeks home-based application)   - questionnaires - pressure pain thresholds - Interviews |
| **Interventions:** | - **Intervention:** Cupping massage   Patients will be introduced to cupping massage by an experienced trainer. First the back is covered on oil, after attaching the cup to the skin by using the rubber ball to induce negative pressure; the cup is moved along the skin. The application will cause hyperaemisation of the skin which will lead to relaxation of the muscles. After application there might be transitory haematoma. All patients are advised to use intervention twice weekly for 12 weeks.   - **Control:** Relaxation   Patients will be introduced to progressive muscle relaxation by an experienced diploma psychologist. With the use of a CD they will practise PMR twice weekly for 12 weeks. |
| **Outcomes:** | Primary outcome   - Change of pain intensity (VAS) from T1 toT2   Secondary outcomes   - Pain and medication according to daily log - Pain at motion (PRTM) - Neck pain disability (NDI) - Anxiety and depression (HADS) - Pain quality (SBL) - Well-being (FEW16) - Stress perception (PSQ20) - Quality of life (SF-36) - Locus of control (GKÜ) - Pressure pain thresholds (PPT)   Exploratory   - Experiences with intervention, safety |
| **Statistics** | Absolute Changes from T1 (Pre) to T2 (Post-Treatment) (T2); Multiple Imputation of missing values; Intention-to-treat-analysis using univariate analysis of covariance (Baseline and expectation as linear Covariates, group as classified Covariate) |
| **Principle investigator** | Dr. med. Thomas Rampp |
| **Scientific Cooperation** | Dr. med. Rainer Stange |
| **Study Coordination** | Dipl.-Psych. Romy Lauche |
| **Statistics** | Dipl.-Stat. Rainer Lüdtke |
| **Sponsor** | - Chair for Complementary and Integrative Medicine - Karl und Veronica Carstens-Foundation, Am Deimelsberg 36, 45276 Essen, Phone +49 201-56305-0: Funding for consumables - Weleda AG, Möhlerstraße 3, 73525 Schwäbisch Gmünd, Phone +49 7171 919 488: free provision of massage oils |

1. Summary

This study aims to test, how effective a home-based cupping massage is compared to a relaxation technique in patients with chronic neck pain. Therefore pain intensity, associated disability, quality of life, well-being and pressure pain sensitivity will be measured in 84 patients with chronic non-specific neck pain. Experiences with the application, safety and [susceptibility](http://www.dict.cc/englisch-deutsch/susceptibility.html) to [infection](http://www.dict.cc/englisch-deutsch/infection.html) will also be documented.

1. Background

Cupping is an ancient therapy which is used regularly in complementary medicine. Traditional concepts claim that cupping, i.e. applying negative pressure to the skin, eliminates toxins. More modern concepts see cupping as one of many therapies where besides hyperaemisation other neurophysiological changes might be induced (Musial, Michalsen, & Dobos, 2008). On the one hand cupping may stimulate mechanosensitive receptors in the treated area. This stimulation might inhibit pain information transmission at the spinal cord neurons. On the other hand, hyperaemisation might change the nociceptors environment. A painful stimulation outside the pain site might also induce the so-called diffuse noxious inhibitory control (DNIC), a supraspinal mechanism that inhibits pain processing.

Different cupping techniques are available. In cupping massage, cupping glasses are placed on the skin. Removing the air from the glass leads to a negative pressure, which causes a fontanel that contains petechial blood and lymph. This process then is expanded by moving the cup over the oiled skin. This method is mainly used at the back muscles and it increases microcirculation, relaxes muscles and eases tension. Locally this might cause a hematoma, however this is transitory.

A typical indication for cupping massage is neck pain, if the pain is caused by tension of the neck muscles (Abele, 2003; Chirali, 2007). Besides cupping massage, dry cupping or wet cupping may also apply. Which technique is indicated, depends in the symptoms and the patient’s physique.

Neck pain is very common, according to the Robert Koch-Institute 56 % of men and 62% of women suffer at least once a year from back pain. 36% of them report pain in the upper back and neck area. Most common causes, besides structural changes and disc prolapses, are muscle tension caused by extensive working in sitting positions and postural deficits (Binder, 2007; Skov, Borg, & Orhede, 1996) in many cases stress seems also to cause and negatively influence neck pain (Linton, 2000). Muscle tension decreases blood flow and microcirculation (Langevin & Sherman, 2007; Larsson, Oberg, & Larsson, 1999) and besides pain patients also report strong impairment such as stiffness. The pain might further radiate, patients with chronic neck pain also show signs of increased pain sensitivity in the affected and adjacent areas (La Touche et al., 2010; Scott, Jull, & Sterling, 2005).

In many pain conditions it is noticeable that patients do not contribute actively to the therapy. They are convinced that only medication might help to handle the pain. That way they overlook their own possibilities to influence the pain themselves. But self-efficacy is very important, as results from a former study indicate (protocol no. 09-3985), because interviews revealed that the perception of having no control might result in helplessness and anger. As a result stress and tension are increased which themselves increase pain perception (Lauche, Cramer, Haller et al., 2011) Therefore it is of utmost importance to teach patients self-help strategies..

Cupping massage might be such a technique that is easily taught. Experiences with courses offered at our department showed high attendance and satisfaction. Cupping massage can easily be used at home and it might increase well-being by release of muscle tension and increased microcirculation. It is also claimed that cupping massage has immune-modulatory effects.

A first pilot study showed positive effects of cupping in chronic neck pain (Lauche, Cramer, Choi et al., 2011).

1. Aim of the study

In this pilot trial the effects of 12 weeks of home-based cupping massage on chronic neck pain will be tested. Outcomes are pain intensity, disability, wellbeing and physiological changes in pain processing.

1. Study design


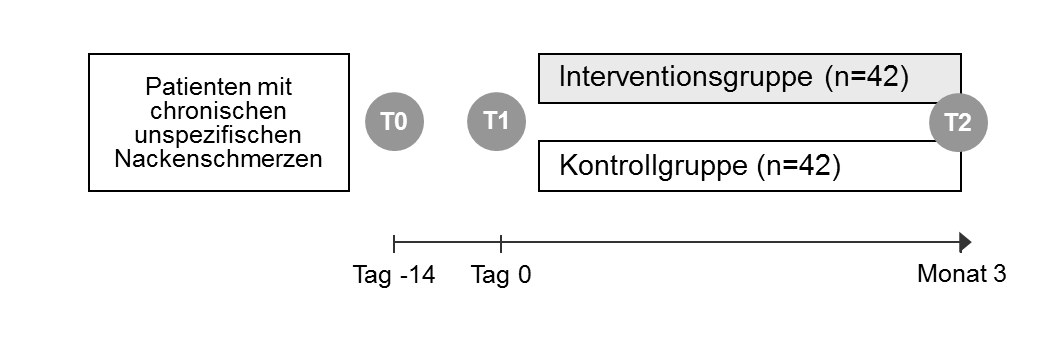


Figure 1: Study design.

The first examination takes place at T0. The second examination and the final inclusion into the study will be performed at T1, at this time patients also fill in the questionnaires and judge their expectation on the effectiveness of both therapies. After the measurement patients will be randomised into one of the two groups. The cupping group will then participate in a workshop on cupping massage; the other group will participate in a workshop for progressive muscle relaxation. During the 12 weeks application all patients will be able to contact the coordinator via telephone in case of problems or questions. A phone call will also be placed after 6 weeks to check for compliance. After 12 weeks of home-based application patients return for T2, the final measurement. An overview over parameters and measurement time points are shown in table 1.

1. Patients
   1. Recruitment

Patients will be recruited via calls on the website of the department and via local press.

- 1. Inclusion criteria

Inclusion criteria are:

- Age between 18 and 75 years
- chronic non-specific neck pain for at least 3 months with intensity > 45mm VAS (Jensen, Chen and Brugger 2003)
- Partner for administration of cupping massage (family, friends)
- Medical records for anamnesis
  1. Exclusion criteria

Participation is not possible in following cases:

- - Specific causes for neck pain, such as :
- Inflammatory diseases
- Neurological disorders
- Disc prolapse
- Trauma, e.g. whiplash, fractures, operation at the upper spine)
- Neoplasms of the spine
- Congenital spine malformation (except for slight scoliosis)
- Skin disease/inflammation/allergic reactions at the areas to be treated
- Haemophilia, anticoagulant medication
- Severe psychiatric disorder
- Severe comorbid disorder (e.g. diabetes mellitus with neuropathy, oncological disease)
- Long-time medication with corticosteroids >10mg Prednisolon-equivalent
- Opiate medication
- Recently started (within the past 6 weeks) or recently modified medication with influences on musculoskeletal pain, such as muscle relaxants, psychopharmacological
- Pregnancy
- Current participation in other clinical studies
  1. Examination
     1. Screening

Patients will be screened telephonically for inclusion and exclusion criteria. If eligible they will be invited for physical examination into the clinic.

- - 1. First round of examination

At T0 a physical examination, an orienting neurological anamnesis will be conducted by the study physician. All patients are asked to provide medical records. At T0 they are informed about the study in detail and they have the possibility to ask the study physician any question. Before they are included they have to provide written informed consent. The data of those patients, that do not meet the criteria, are also documented.

- - 1. Second round of examination

After the first examination all patients will be handed out a daily log, which was checked at the second examination. Only patients who regularly filled in the daily log (>80% of days) and whose pain on average was >45mm VAS, were randomized for the study.

- 1. Randomization

After the measurement at T1 all patients will be randomized into one of the two groups using a non-stratified block randomization with random varying block lengths. The statistician will draw random numbers using the random number generator ranuni“ from the SAS®-Software (release 9.2, SAS Inc., Cary NC, USA) and prepare sealed numbered opaques sorted in ascending order. As soon a patient is included into the study the opaque with the lowest number will be opened and the patient assigned to the stated group.

1. Intervention

All intervention will be performed at the patients’ home. Patients receive extensive training, written material as well as consumables (cupping glass, oil, CD for relaxation). For questions patients could contact the coordinator or physician at any time during the study.

- 1. Cupping massage

Patients in that group participate in a workshop. It will be conducted by an experienced cupping therapist and accompanied by a physician or a psychologist. It will introduce history and background of cupping massage, indications, contraindications as well as the technique. Subsequently all patients will be provided with the material and after a short demonstration, all patients will exercise the technique with their partners under supervision. Supervisors will especially response to the individual complaints of the patients. After the workshop patients take home an information sheet, the cupping glass and massage oil. During the whole study patients will be able to contact the coordinator via telephone; this should reduce drop outs and increase compliance.

Cupping massage will be conducted on intact skin. As preparation the skin will be oiled with arnica massage oil (Weleda), then the cupping glass will be attached on the skin and negative pressure will be created using the rubber ball at the top of the glass. The cup will be drawn over the skin along the muscles of the back, from the occiput towards the midlevel thoracic spine as well as over the upper trapezius muscle. Pain areas will be treated intensively. Along treated areas petechiae might occur. The cupping massage takes about 10 minutes and after the massage patients are advised to rest. Study patients should use cupping massage twice weekly for 12 weeks, favourably in the evening hours.

- 1. Relaxation training

Patients in that group participate in a workshop on Progressive Muscle relaxation (PME). A diploma psychologist teaches the history, the background and the practise of PME, and then the patients will practise the PME under supervision. Afterwards all questions are answered and patients are able to share their experiences. At the end, all patients receive a CD (Techniker Krankenkasse) to practise PME at home twice weekly for 12 weeks. During the whole study patients will be able to contact the coordinator via telephone; this should reduce drop outs and increase compliance. After the study patients in that group are offered to participate in a cupping massage workshop and they also receive the cupping glass and the massage oil for free.

1. Methods

An overview over parameters and measurement time points are shown in table 1.

| **Parameters** | **T0** | **T1** | **T2** |
| --- | --- | --- | --- |
| Anamnesis, Socio-demographic data | X |  |  |
| Expectation of therapies |  | X |  |
| Questionnaires :  Neck pain intensity (VAS), Pain at motion (PRTM), pain quality (SBL) Disability (NDI), Well-being (FEW16), Anxiety and Depression (HADS), Stress perception (PSQ20), quality of life (SF-36), locus of control beliefs (GKÜ) |  | X | X |
| Pressure pain sensititiviy (PPT) |  | X | X |
| Daily log (Drugs, pain intensity, concurrent therapies) | X | X | X |
| Safety |  |  | X |
| Interviews |  |  | X |

- 1. Measurement instruments

At first patient specific data (socio-demographic data as age, gender, hight, weight, socioeconomic data), data on neck pain, previous therapies and medication are gathered. In a daily log, patients fill in daily pain intensity on a visual analogue scale, the use of medication and other concurrent therapies during the study.

Further standardised questionnaires will be used:

- Pain intensity (visual analogue scale, **VAS, 0-100mm**)
- Pain at motion (**PRTM**) (Irnich et al., 2001). This questions determine pain induced by head movement in six movement directions. The pain for each movement is measured by a visual analogue scale - VAS (0-100mm).
- Disability (**NDI**) (Vernon & Mior, 1991). 10 Items determine the impact of neck pain on the everyday activities.
- Pain quality (**SBL**) (Korb & Pfingsten, 2003). With the 12-Item short version of the pain perception scale the perception of sensory and affective dimensions of pain are determined.
- Anxiety and Depression (**HADS**) (Herrmann, Buss, & Snaith, 1995).
- Quality of life (**SF-36**) (Bullinger & Kirchberger, 1998). The SF-36 measures health related quality of life on 8 dimensions and 2 summary component scales. For the SF-36 norm values are available for comparison.
- Locus of control beliefs (**GKÜ**) (Hasenbring, 1989). This questionnaire evaluates the patients beliefs, who or what circumstances can influence the disease.
- Well-being (**FEW16**) (Kolip & Schmidt, 1999). With 16 items this questionnaire measures 4 dimensions of habitual physical wellbeing.
- Stress perception (**PSQ20**) (Fliege et al., 2005). The perceived stress independent from specific situations is measured with the PSQ 20.

Pressure pain thresholds will be determined using a Algometer from Somedic, as it was used in prior studies (protocol numbers 09-3895, 09-3896, 09-3987). Pressure will be increased in ramps of 40kPa/s until the patient reports pain additionally to pressure. The pressure measured at this moment will be documented and 3 measures will be averaged. The PPT will be measured at the site of maximal pain and bilaterally on defined muscles (levator scapu­lae muscle, semispinalis capitis muscle, trapezius muscle). The PPT at the right hand (Thenar) will serve for demonstration and estimation of reliability (Johnston, Jimmieson, Jull, & Souvlis, 2008).

After the intervention patients’ experiences with application, compliance and observed changes will be retrieved in interviews. Safety will also be measured.

The influence of application on partnership and the susceptibility to infection will be part of the interviews. They might provide information for further studies.

- 1. Measurement time points

The parameters are measured before intervention (T1) and post intervention (T2). No long term catamnesis will be included as patients in the relaxation group will also receive cupping at the end of the study.

The daily log will be used during the whole study. The seminstandardized interview will only be conducted at post-intervention (T2).

1. Outcomes
   1. Primary outcome

Change in pain intensity from T1 to T2

- 1. Secondary outcome
- Change in pain intensity, medication and concurrent therapies according to the daily log
- Pain at motion (PRTM)
- Neck pain specific disability (NDI)
- Pain quality (SBL)
- Well-being (FEW16)
- Anxiety and depression (HADS)
- Stress perception (PSQ20)
- Quality of life (SF-36)
- Locus of control beliefs (GKÜ)
- Pressure pain sensitivity (PPT)
  1. Exploratory outcomes

Experiences in application, compliance and observed changes during the study will be measured descriptively. Safety will be measured the same way.

The influence of cupping massage on partnership will be measured using semi-standardised interviews. The analysis will be conducted using the qualitative content analysis approach after Mayring (Mayring, 2008).

1. Statistics
   1. Estimation of required sample size

A literature search revealed no interventional study on which the estimation could be based on. Therefore data of a non-reported trial (protocol no. 09-3987) were used. Analysis revealed a difference of -14.3mm on the VAS the effect size was d=0.66. This effect size was used for estimation, although the settings of the planned study were different from the prior study (application at home instead of in the clinic, longer treatment intervals, empathic partner instead of physician), which made it impossible to determine the effect to be expected.

Based on the effect size of 0.66, an α of 5% and a power of 80%, a two-sided t-test would require 38 patients per group, i.e. 76 per study. To compensate for 10% drop outs, 84 patients were to be included in the study.

- 1. Statistical analysis

All analyses are based on the intention to treat population, i.e. all randomized patients. Missing values are to be imputed using multiple Imputation technic (MCMC-Method). Altogether 50 complete data sets are generated that way (Procedure PROC MI der SAS/STAT®-Software), analysed and the differences will be averaged (Procedure PROC MIANALYZE).

The primary outcome will be analyses using analysis of covariance model, with group as binary covariate, baseline and expectation as linear covariates. An adjusted group difference will be estimated (incl. 95% confidence intervals) and the superiority of cupping will be tested using a two-sided t-test.

No interims analyses are planned. No adjustment of results is necessary.

For the secondary outcomes equal models will be used for analyses. Group differences and significance will only be interpreted descriptively.

1. Risk Benefit Analysis
   1. Cupping massage

Cupping massage is considered very safe and used routinely in the department for Complementary and Integrative Medicine. Patients were instructed in cupping massage for years now, and the feedback was positive. If used after correct anamnesis and in the absence of contraindications no risks have been reported. Formation of haematoma is transitory.

- 1. Relaxation

Relaxation exercises are often used in stress-associated diseases. The effects are mostly unspecific, no side effects are known.

- 1. Measurements

No risks are expected to arise from questionnaires. The measurement of the pressure pain thresholds are validated and increased pain is only expected under pathological conditions. The measurement will be interrupted as soon as the patients perceive first signs of pain, therefore the risk of injury are very small.

- 1. Summary of Risks and benefits

Cupping massage is used at the department of Internal and Integrative medicine with great success. The first pilot study (protocol no 09-3987) shows promising results. In sight of many years of practice and the easy handling of cupping massage, it can be assumed that the benefits exceed the risks.

1. Study protocol

The principle investigator will submit the study protocol to the ethics commission. The study will be started after approval of the ethics commission. The ethics commission will be informed in cases of deviations from the protocol, serious or unknown adverse events or new results indicating a threat to patients’ safety.

1. Insurance

No patient insurance policy is taken out, there will also be no insurance for commuting accidents. All patients will be made aware of that.

1. Patient information, informed consent and data protection

According to § 4(1) BDSG (Bundesdatenschutzgesetz) the collection, processing and use of personalized data is only permitted if the person concerned has agreed. In order to anonymise the data, all questionnaires will be numbered using a numerical code.

Participating patients will be informed about the study, the background, the risks and their rights, especially the right to resign from the study at any time point. Patients receive a written information and they have the possibility to counsel the study investigator/physician about the study conditions. Patients can withdraw their consent at any time without any disadvantages. In case of withdrawal of their consent all data of that person will be deleted.

The participant will be informed about the aim, the collected data and the use of personalized data. Patients who do not give their consent cannot be allocated.

Participants will be informed that data, if necessary, will be

a) used for Inspection of the proper conduction of the trial

b) send to the principle investigator and the statistician for analysis - pseudonymised

c) published in scientific form - anonymised

1. Financing and conflict of interest

The study will be sponsored by the chair of Complementary and integrative Medicine. Further did the principle investigator receive a funding from the Karl and Veronica Carstens foundation (Karl und Veronica Carstens-Stiftung, Am Deimelsberg 36, 45276 Essen, Telephone +49 201-56305-0, www.carstens-stiftung.de) that covers the cost for consumables and the costs for the workshop leader. The WELEDA company (Weleda AG, Möhlerstraße 3, 73525 Schwäbisch Gmünd, Phone +49 7171 919 488, Telefax +49 7171 919 87 488, http://www.weleda.de) is providing the massage oils for free. None of the sponsors has any other role in any stage of the study.

1. Termination of study

The study will be closed after recruitment of 84 patients. It can be terminated prematurely if patients are not recruited within 6 months, if serious deviations of the protocol occur, if documentation is filled out flawed or intentionally flawed or if legal and ethical regulations are violated.

The premature termination is to be decided by the principle investigator and the physicians. If serious adverse events are accumulating the principle investigator may decide termination on his own.

The study ends for each patient after the post-intervention measurement or when the consent is withdrawn. The participation can further be ended in case of serious adverse event, the occurrence of a severe disease or the non-compliance if this increases the probability of adverse events.

1. Eligibility of trial site

The clinical trial facility has years of experience in conducting clinical trials. It also has experienced personal, laboratories and computer work spaces.

1. References

Abele, J. (2003). *Das Schröpfen: eine bewährte alternative Heilmethode* (5. ed.). München: Urban & Fischer Verlag.

Binder, A. (2007). The diagnosis and treatment of nonspecific neck pain and whiplash. *Europa Medicophysica, 43*(1), 79-89.

Bullinger, M., & Kirchberger, I. (1998). *SF-36. Fragebogen zum Gesundheitszustand. Handanweisung*. Göttingen: Hogrefe.

Chirali, I. (2007). *Traditional chinese medicine cupping therapy* (2nd ed.). Philadelphia, PA: Elsevier Churchill Livingston.

Fliege, H., Rose, M., Arck, P., Walter, O. B., Kocalevent, R. D., Weber, C., et al. (2005). The perceived stress questionnaire (PSQ) reconsidered: Validation and reference values from different clinical and healthy adult samples. *Psychosomatic Medicine, 67*(1), 78-88.

Hasenbring, M. (1989).
Laienhafte ursachenvorstellungen und erwartungen zur Beeinflußbarkeit einer krebserkrankung - erste ergebnisse einer studie an krebspatienten. In C. Bischoff, & H. Zenz (Eds.), *Patientenkonzepte von körper und krankheit* (pp. 25-38). Bern: Huber.

Herrmann, C., Buss, U., & Snaith, R. P. (1995). *Hospital Anxiety and Depression Scale - Deutsche Version (HADS-D). Manual*. Bern: Hans Huber.

Irnich, D., Behrens, N., Molzen, H., Konig, A., Gleditsch, J., Krauss, M., et al. (2001, Jun 30). Randomised trial of acupuncture compared with conventional massage and "sham" laser acupuncture for treatment of chronic neck pain. *BMJ (Clinical Research Ed.), 322*, 1574-1578.

Jensen, M. P., Chen, C., & Brugger, A. M. (2003). Interpretation of visual analog scale ratings and change scores: A reanalysis of two clinical trials of postoperative pain. *The Journal of Pain : Official Journal of the American Pain Society, 4*(7), 407-414.

Johnston, V., Jimmieson, N. L., Jull, G., & Souvlis, T. (2008). Quantitative sensory measures distinguish office workers with varying levels of neck pain and disability. *Pain, 137*(2), 257-265.

Kolip, P., & Schmidt, B. (1999). Der Fragebogen zur Erfassung körperlichen Wohlbefindens (FEW16). Konstruktion und erste Validierung. *Zeitschrift für Gesundheitspsychologie, 7*, 77-87.

Korb, J., & Pfingsten, M. (2003). Der deutsche schmerzfragebogen - implementierte psychometrie. *Schmerz, 17*, S47.

La Touche, R., Fernandez-de-Las-Penas, C., Fernandez-Carnero, J., Diaz-Parreno, S., Paris-Alemany, A., & Arendt-Nielsen, L. (2010). Bilateral mechanical-pain sensitivity over the trigeminal region in patients with chronic mechanical neck pain. *The Journal of Pain : Official Journal of the American Pain Society, 11*(3), 256-263.

Langevin, H. M., & Sherman, K. J. (2007). Pathophysiological model for chronic low back pain integrating connective tissue and nervous system mechanisms. *Medical Hypotheses, 68*(1), 74-80.

Larsson, R., Oberg, P. A., & Larsson, S. E. (1999). Changes of trapezius muscle blood flow and electromyography in chronic neck pain due to trapezius myalgia. *Pain, 79*(1), 45-50.

Lauche, R., Cramer, H., Choi, K. E., Rampp, T., Saha, F. J., Dobos, G. J., et al. (2011). The influence of a series of five dry cupping treatments on pain and mechanical thresholds in patients with chronic non-specific neck pain - a randomised controlled pilot study. *BMC Complementary and Alternative Medicine, 11*(1), 63.

Lauche, R., Cramer, H., Haller, H., Musial, F., Langhorst, J., Dobos, G. J., et al. (2011). “My body shrinks” – the influence of traditional cupping on the body image in patients with chronic non-specific neck pain. *Journal of Traditional Chinese Medicine, 31*(Supplement), 46-46.

Linton, S. J. (2000). A review of psychological risk factors in back and neck pain. *Spine, 25*(9), 1148-1156.

Mayring, P. (2008). *Qualitative Inhaltsanalyse. Grundlagen und Techniken* (10. Auflage ed.). Weinheim und Basel: Beltz.

Musial, F., Michalsen, A., & Dobos, G. (2008). Functional chronic pain syndromes and naturopathic treatments: Neurobiological foundations. *Forschende Komplementarmedizin (2006), 15*(2), 97-103.

Scott, D., Jull, G., & Sterling, M. (2005). Widespread sensory hypersensitivity is a feature of chronic whiplash-associated disorder but not chronic idiopathic neck pain. *The Clinical Journal of Pain, 21*(2), 175-181.

Skov, T., Borg, V., & Orhede, E. (1996). Psychosocial and physical risk factors for musculoskeletal disorders of the neck, shoulders, and lower back in salespeople. *Occupational and Environmental Medicine, 53*(5), 351-356.

Vernon, H., & Mior, S. (1991). The neck disability index: A study of reliability and validity. *Journal of Manipulative and Physiological Therapeutics, 14*(7), 409-415.
